# Chromosomal-level assembly of the blood clam, *Scapharca* (*Anadara*) *broughtonii*, using long sequence reads and Hi-C

**DOI:** 10.1093/gigascience/giz067

**Published:** 2019-07-09

**Authors:** Chang-Ming Bai, Lu-Sheng Xin, Umberto Rosani, Biao Wu, Qing-Chen Wang, Xiao-Ke Duan, Zhi-Hong Liu, Chong-Ming Wang

**Affiliations:** 1 Key Laboratory of Maricultural Organism Disease Control, Ministry of Agriculture; Laboratory for Marine Fisheries Science and Food Production Processes, Qingdao National Laboratory for Marine Science and Technology; Qingdao Key Laboratory of Mariculture Epidemiology and Biosecurity; Yellow Sea Fisheries Research Institute, Chinese Academy of Fishery Sciences, 106 Nanjing Road, Qingdao 266071, China; 2 Department of Biology, University of Padua, Via Ugo Bassi 58/B, Padua 35121, Italy; 3 Alfred Wegener Institute – Helmholtz Centre for Polar and Marine Research, Wadden Sea Station, Hafenstraße 43, List/Sylt 25992, Germany; 4 Biomarker Technologies Corporation, 12 Fuqian Street, Beijing 101200, China

**Keywords:** ark shell, PacBio, Hi-C, genomic, chromosomal assembly

## Abstract

**Background:**

The blood clam, *Scapharca* (*Anadara*) *broughtonii*, is an economically and ecologically important marine bivalve of the family Arcidae. Efforts to study their population genetics, breeding, cultivation, and stock enrichment have been somewhat hindered by the lack of a reference genome. Herein, we report the complete genome sequence of *S. broughtonii*, a first reference genome of the family Arcidae.

**Findings:**

A total of 75.79 Gb clean data were generated with the Pacific Biosciences and Oxford Nanopore platforms, which represented approximately 86× coverage of the *S. broughtonii* genome. *De novo* assembly of these long reads resulted in an 884.5-Mb genome, with a contig N50 of 1.80 Mb and scaffold N50 of 45.00 Mb. Genome Hi-C scaffolding resulted in 19 chromosomes containing 99.35% of bases in the assembled genome. Genome annotation revealed that nearly half of the genome (46.1%) is composed of repeated sequences, while 24,045 protein-coding genes were predicted and 84.7% of them were annotated.

**Conclusions:**

We report here a chromosomal-level assembly of the *S. broughtonii* genome based on long-read sequencing and Hi-C scaffolding. The genomic data can serve as a reference for the family Arcidae and will provide a valuable resource for the scientific community and aquaculture sector.

## Background

**Figure 1:**
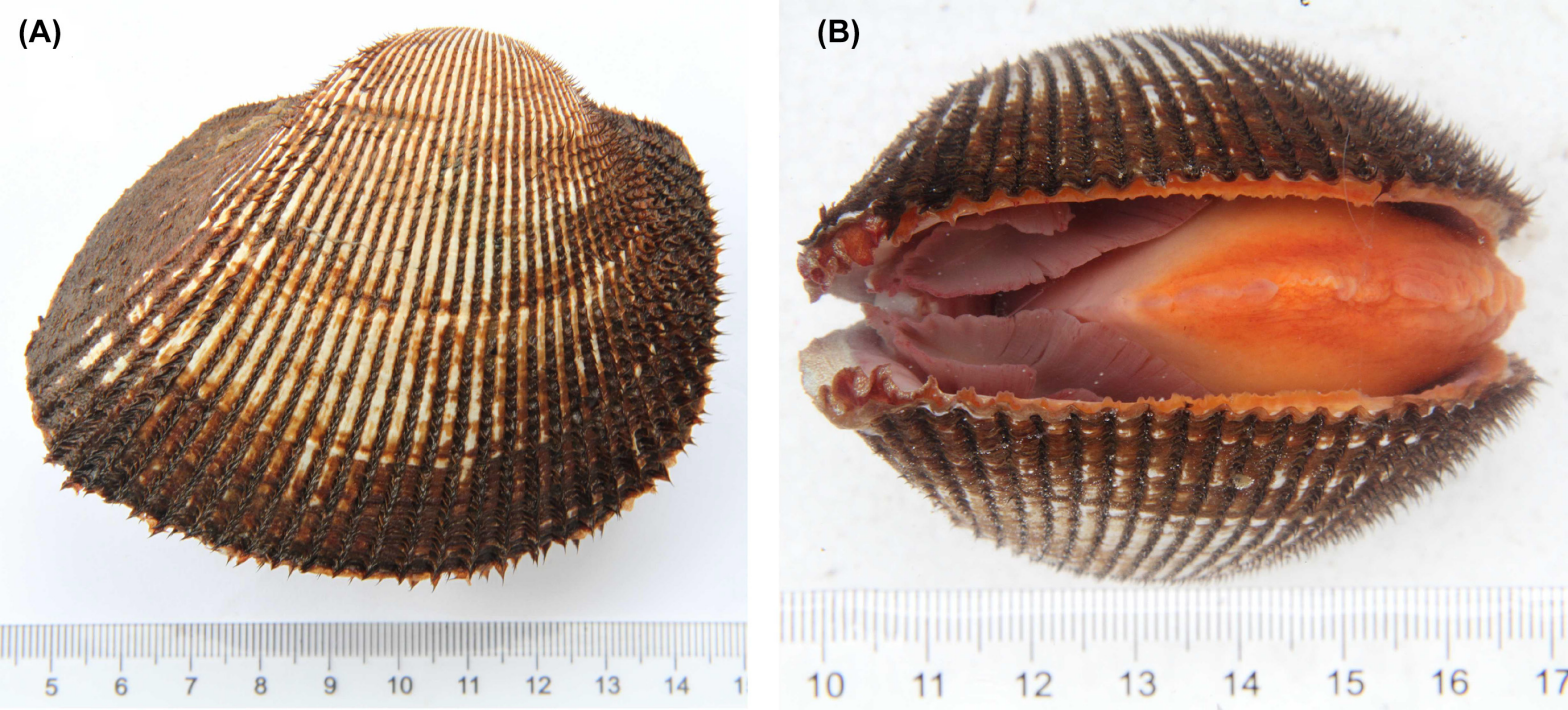
Example of a *Scapharca* (*Anadara*) *broughtonii*, the blood clam.

The blood clam, *Scapharca* (*Anadara*) *broughtonii* (Schrenck, 1867; NCBI:txid148819; marinespecies.org: taxname:504357), is a species of ark shell of the family Arcidae, class Pteriomorphia, phylum Mollusca. Although most of the approximately 200 species of the family Arcidae are distributed in tropical areas [1], *S. broughtonii* lives in temperate areas along the coasts of Northern China, Japan, Korea, and the Russian Far East [1, 2]. The name “blood clam” originated from the red color of their visceral mass, which is due to the presence of hemoglobin in both tissues and hemolymph [1, 2], a rare trait in molluscs but a hallmark of Arcidae species [3]. *S. broughtonii* specimens are characterized by thick and harder calcareous shells, covered by a hairy brown periostracum (Fig. 1) [2]. Adult blood clams can reach a shell length of 100 mm [4] and are harvested as a source of sashimi, which has contributed to the depletion of wild resources in the past century. Many efforts have been made to recover the wild population stocks of *S. broughtonii* in China, Japan, and Korea, including intensive farming. Such aquaculture practices have revealed the susceptibility of *S. broughtonii* to many pathogenic bacteria and viruses, including a variant of the *Ostreid herpesvirus 1* [1, 5–7]. Compared with other aquaculture-important bivalve species, such as oysters, mussels, and scallops, the genomic and transcriptomic resources of Arcidae species are still limited. Therefore, the understanding of their basic biological processes, as well as of more complex host-pathogen interactions, is somewhat hampered. Here, we sequenced the complete genome of *S. broughtonii* at the chromosomal level and we offer it as a valuable resource to develop both scientific research and aquaculture industry related to Arcidae species.

## Sample Collection and Sequencing

Adult *S. broughtonii* specimens were sampled from populations near Jimo, Shandong Province, China. To overcome the excessive polysaccharide content of *S. broughtonii* tissues, high-quality genomic DNA was extracted from hemocytes, using DNeasy^®^ Blood & Tissue Kit (Qiagen, Hilden, Germany, p/n 69,504) with a few protocol modifications to remove polysaccharides (the detailed protocol is reported at protocols.io [8] and Supplementary Table S1). The DNA quantity and quality were measured with Qubit 3.0 (Thermo Fisher Scientific, Inc., Carlsbad, CA, USA) and agarose gel electrophoresis, respectively. High-quality DNA was used for library preparation and high-throughput sequencing using PacBio, Nanopore, and Illumina platforms (Table   1, BioMarker Technology Co. Ltd., Beijing, China).

**Table 1:** Summary of sequencing data generated for blood clam genome assembly and annotation

Library type	Platform	Library size (bp)	Data size (Gb)	Application
Short reads	HiSeq X Ten	350	53.06	Genome survey, correction, and evaluation
Long reads	PacBio SEQUEL	20,000	63.33	Genome assembly
	PacBio RS II	20,000	3.99	
	Nanopore Minion	20,000	8.47	
Hi-C	HiSeq X Ten	350	52.16	Chromosome construction

PacBio sequencing was carried out with the SMRT Bell^TM^ library using a DNA Template Prep Kit 1.0 (Pacific Biosciences [PacBio], Menlo Park, CA, USA, p/n 100–259-100). All the detailed library preparation protocols are available on protocols.io [9]. Briefly, the genomic DNA (10 μg) was mechanically sheared using a Covaris g-Tube (Covaris, Inc., Woburn, MA, USA, p/n 520,079) to get DNA fragments of ∼20 kb in size. The sheared DNA was DNA-damage and end-repaired using polishing enzymes. Then a blunt-end ligation reaction followed by exonuclease treatment was conducted to generate the SMRT Bell^TM^ template. Finally, large fragments (>10 kb) were enriched with Blue Pippin device (Sage Science, Inc., Beverly, MA, USA) for sequencing. A total of 15 single-molecule real-time (SMRT) cells were processed, 7 with Sequel and 8 with RS II instruments (PacBio), to generate a total of 67.32 Gb PacBio data. For Oxford Nanopore sequencing, ∼5 μg of genomic DNA was sheared and size-selected (∼20 kb) with the aforementioned procedure. The selected fragments were processed using the Ligation Sequencing 1D Kit (Oxford Nanopore, Oxford, UK, p/n SQK-LSK109) according to the manufacturer's instructions and sequenced using the MinION portable DNA sequencer with the 48 hours run script (Oxford Nanopore), to generate a total of 8.47 Gb data. For Illumina sequencing, a paired-end (PE) library with an insert size of 350 bp was constructed in accordance with the manufacturer's protocol, and sequenced with an Illumina HiSeq X Ten platform (Illumina, Inc., San Diego, CA, USA) with paired-end 150 (PE150) read layout. A total of 53.06 Gb Illumina data were generated and used for genome survey, correction, and evaluation (Supplementary Table S2). All high-throughput sequencing data have been deposited at the NCBI SRA database under accession ID SAMN10879241.

## Initial Genome Assembly and Evaluation

The Sequel and RS II raw files (bam and H5 formats) were converted into subreads in fasta format with the standard PacBio SMRT software package, for a total of 63,330,577,481 and 3,990,849,516 bp, respectively. Subreads shorter than 500 bp in size were filtered out, to obtain a clean dataset of 4,761,097 PacBio reads for a total of 67,260,156,459 bp, with a read N50 of 21,932 and a mean read length of 14,127 bp (Supplementary Table S3). The Nanopore reads were base-called from the raw FAST5 files using Guppy implanted in MinKNOW (Oxford Nanopore), applying a minimum length cut-off of 500 bp, for a total of 8,468,912,896 bp, with a read N50 of 20,804 and a read mean length of 15,143 bp (Supplementary Table S4). Hybrid assembly of the clean reads was carried out using Canu v1.5 (Canu, RRID:SCR_015880) [10] and WTDBG v1.1 [11] tools. The 2 assemblies were joined using Quickmerge v0.2.2 [12], and the redundancy was removed with Numer v4.0.0 [13]. Finally, the genome assembly was corrected for 3 cycles with the Illumina reads prepared specifically for genome survey using Pilon v1.22 (Pilon, RRID:SCR_014731) with default settings [14]. This initial genome assembly was 884,500,940 bp in length with a contig N50 of 2,388,811 bp (Supplementary Table S5). The detailed parameters of each tool used for genome assembly are available at protocols.io [15].

We evaluated the quality of the initial assembly by mapping the 360,937,442 Illumina reads for genome survey to the assembly using SAMTools v0.1.18 (SAMTOOLS, RRID:SCR_002105) [16] and by searching the 303 eukaryotic and 978 metazoan conserved genes in the assembly using BUSCO v2.0 (BUSCO, RRID:SCR_015008) [17]. As a result, 97.45% of the Illumina reads were successfully mapped to the assembled genome. The BUSCO analysis found 273 and 897 conserved genes belonging to eukaryote and metazoan datasets, accounting for 90.10% and 91.72% of the totals, respectively (Supplementary Table S6). These results indicated the considerable quality of this initial genome assembly of *S. broughtonii*.

## Hi-C Analysis and Chromosome Assembly

Fresh adductor muscle collected from a single *S. broughtonii* specimen of the same population was first fixed using formaldehyde with a final concentration of 1%. The fixed tissue was then homogenized with tissue lysis, digested with the restriction enzyme (HindIII), *in situ* labeled with a biotinylated residue, and end-repaired. Finally, the DNA was extracted and used for Hi-C library preparation using the Nextera Mate Pair Sample Preparation Kit (Illumina, p/n FC-132–1001). Briefly speaking, 5–6 μg DNA was first sheared, end-repaired, and selected for fragments with a length of 300–700 bp, and the biotin-containing fragments were captured. Then the basic standard steps of dA-tailing, adapter ligation, PCR amplification, and purification were carried out. Finally, the quality of the purified library was evaluated with Qubit 3.0 (Thermo Fisher Scientific, Inc.), quantitative PCR (Q-PCR), and Caliper LabChip GX Analyzer (Waltham, MA, USA). The qualified library was sequenced using an Illumina HiSeq X Ten platform with 150 PE layout. A total of 174,148,156 read pairs (52.16 Gb) with a Q30 of 93.16% were generated and used for the subsequent Hi-C analysis (NCBI SRA accession number: SAMN10879242).

To get the unique mapped read pairs, the 174 million read pairs were first truncated at the putative Hi-C junctions and then aligned to the *S. broughtonii* genome assembly using the BWA aligner v0.7.10-r789 (BWA, RRID:SCR_010910) [18]. A total of 206 million reads (59.23%) mapped to the assembled genome, of which 51 million read pairs (29.33%) were uniquely mapped (Supplementary Table S7). Only the uniquely aligned pairs with a mapping quality >20 were further considered, while the invalid interaction pairs due to self-circle ligation, dangling ends, re-ligation, and the other dumped types were filtered out with HiC-Pro v2.10.0 [19]. A total of 17 million valid interaction pairs, accounting for 33.66% of the unique mapped read pairs (Supplementary Table S8), were used for the Hi-C analysis. Detailed Hi-C assembly parameters are available at protocols.io [20].

**Figure 2:**
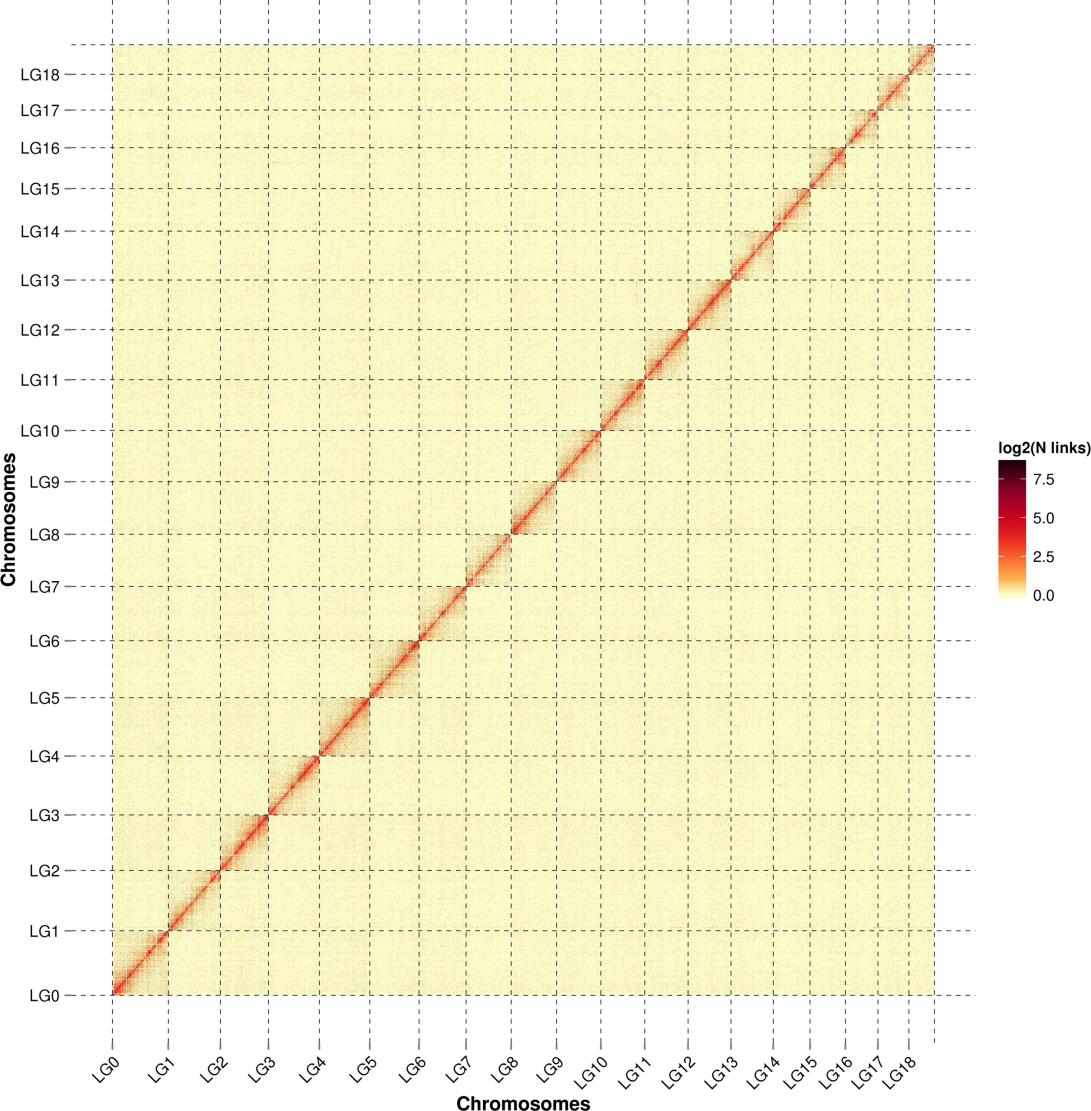
Hi-C interaction heat map for *Scapharca* (*Anadara*) *broughtonii*.

To correct misassemblies that occurred in the initial assembly, the contigs were broken into 300-bp fragments and then assembled on the basis of Hi-C data using Lachesis v2e27abb [21]. The genomic regions characterized by the sudden drop of physical coverage were defined as misassemblies, and contigs were broken at that point [22]. As a result, we identified 343 break points in 156 contigs, and 1,645 corrected contigs with an N50 of 1.81 Mb and a length of 884.50 Mb. Then the corrected contigs were reassembled into 1677 contigs using Lachesis that conbined Hi-C data. Finally, 1,384 contigs (82.53%) were successfully clustered into 19 groups (Fig. 2), which was consistent with previous karyotype analyses of *S. broughtonii* [23]. The 1,384 clustered contigs correspond to a length of 878.79 Mb (99.35% of the length of the corrected contigs). Among the 1,384 clustered contigs, 670 contigs (819.17 Mb) were anchored with defined order and orientation, accounting for 48.41% and 93.22% of the reassembled contigs by contig number and length, respectively (Supplementary Table S9). The final chromosomal-level *S. broughtonii* genome assembly, which represented the first reference genome of Family Arcidae, has a contig N50 of 1.80 Mb and scaffold N50 of 45.00 Mb (Table 2).

**Table 2:** Statistics of the final genome assembly of *Scapharca* (*Anadara*) *broughtonii*

Types	Number	Length (bp)	N50 (bp)	N90 (bp)	Maximum (bp)	Guanine-cytosine content (%)	Gap (bp)
Scaffold	1,026	884,566,040	44,995,656	25,444,477	55,667,740	33.70	65,100
Contig	1,677	884,500,940	1,797,717	305,905	7,852,409	33.70	0

## Genome Annotation

We used LTR FINDER v1.05 (LTR_Finder, RRID:SCR_015247) [24], RepeatScout v1.0.5 (RepeatScout, RRID:SCR_014653) [25], and PILER-DF v2.4 [26] to construct a library of repetitive sequences based on the *S. broughtonii* genome. We classified these repeats using PASTEClassifier v1.0 [27], and we merged them with the Repbase database [28]. Finally, RepeatMasker v4.0.5 (RepeatMasker, RRID:SCR_012954) [29] was used to identify and mask the genomic repeated sequences for a total length of 407.8 Mb, representing 46.1% of the total genome length. The statistics of amount, length, and percentage of each repeat type can be found in Supplementary Table S10. Additional methodological information about genome annotation is available at protocols.io [15].

Protein-coding genes were predicted using the following approaches: *ab initio* prediction, homology-based prediction, and transcriptome-based prediction. For *ab initio* prediction, Genscan v3.1 (Genscan, RRID:SCR_012902) [30], Augustus v3.1 (Augustus, RRID:SCR_008417) [31], GlimmerHMM v1.2 (GlimmerHMM, RRID:SCR_002654) [32], GeneID v1.4 [33], and SNAP v2006–07-28 (SNAP, RRID:SCR_002127) [34] were used. For homology-based prediction, protein sequences of 3 closely related mollusc species (*Crassostrea gigas, Mizuhopecten yessoensis*, and *Mytilus galloprovincialis*) and *Danio rerio* were downloaded from NCBI and aligned against the assembled genome with GeMoMa v1.3.1 [35]. For the transcriptome-based prediction, transcriptomic data obtained from a previous study (NCBI SRA accession ID: PRJNA450478) [36] were used as input data. In the previous study [36], RNA-seq data had been *de novo* assembled with Trinity v.r20140413p1 and the gene predictions were carried out with Program to Assemble Spliced Alignments (PASA) v2.0.2 (PASA, RRID:SCR_014656) [37]. We also performed reference-based assembly of the RNA-seq data with Hisat v2.0.4 (HISAT2, RRID:SCR_015530) and Stringtie v1.2.3 [38], then we predicted the genes using TransDecoder v2.0 [39] and GeneMark v5.1 (GeneMark, RRID:SCR_011930) [40]. All the gene predictions were integrated using EVidenceModeler (EVM) v1.1.1 (EVM, RRID:SCR_014659) [41], and further modified with PASA v2.0.2, to obtain a final dataset of 24,045 predicted genes with an average length of 12,549 bp (Supplementary Table S11).

Pseudogenes emerge from coding genes that have become non-functional due to accumulation of mutations [42, 43]. A sequence that is homologous to a normal protein-coding gene but not annotated as protein-coding genes is likely to be a pseudogene. Therefore, based on homology to known protein-coding genes, putative pseudogenes were first searched in the intergenic regions of the *S. broughtonii* genome using genBlastA v1.0.4 [44]. Then GeneWise v2.4.1 (GeneWise, RRID:SCR_015054) [45] was adopted to search the premature stop codons or frameshift mutations in those sequences and to finally identify a total of 1,658 pseudogenes, with an average length of 3,151 bp.

**Figure 3:**
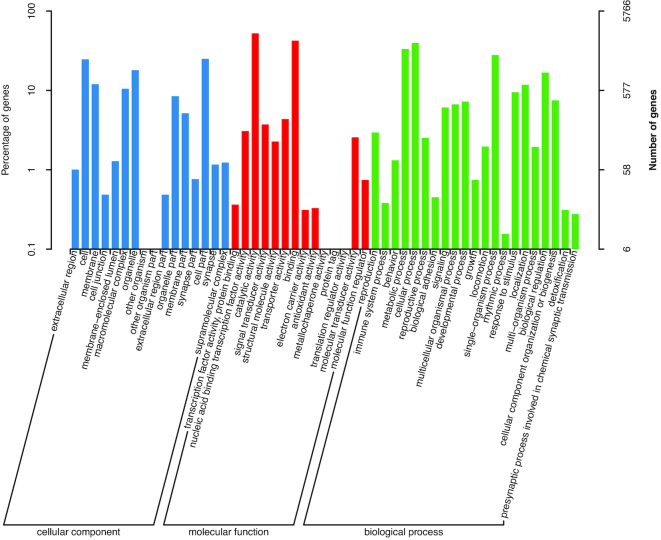
Gene ontology (GO) annotation of the predicted genes. The horizontal axis indicates classes of the second-level GO annotation. The vertical axis indicates the number and percentage of genes in each class.

**Figure 4:**
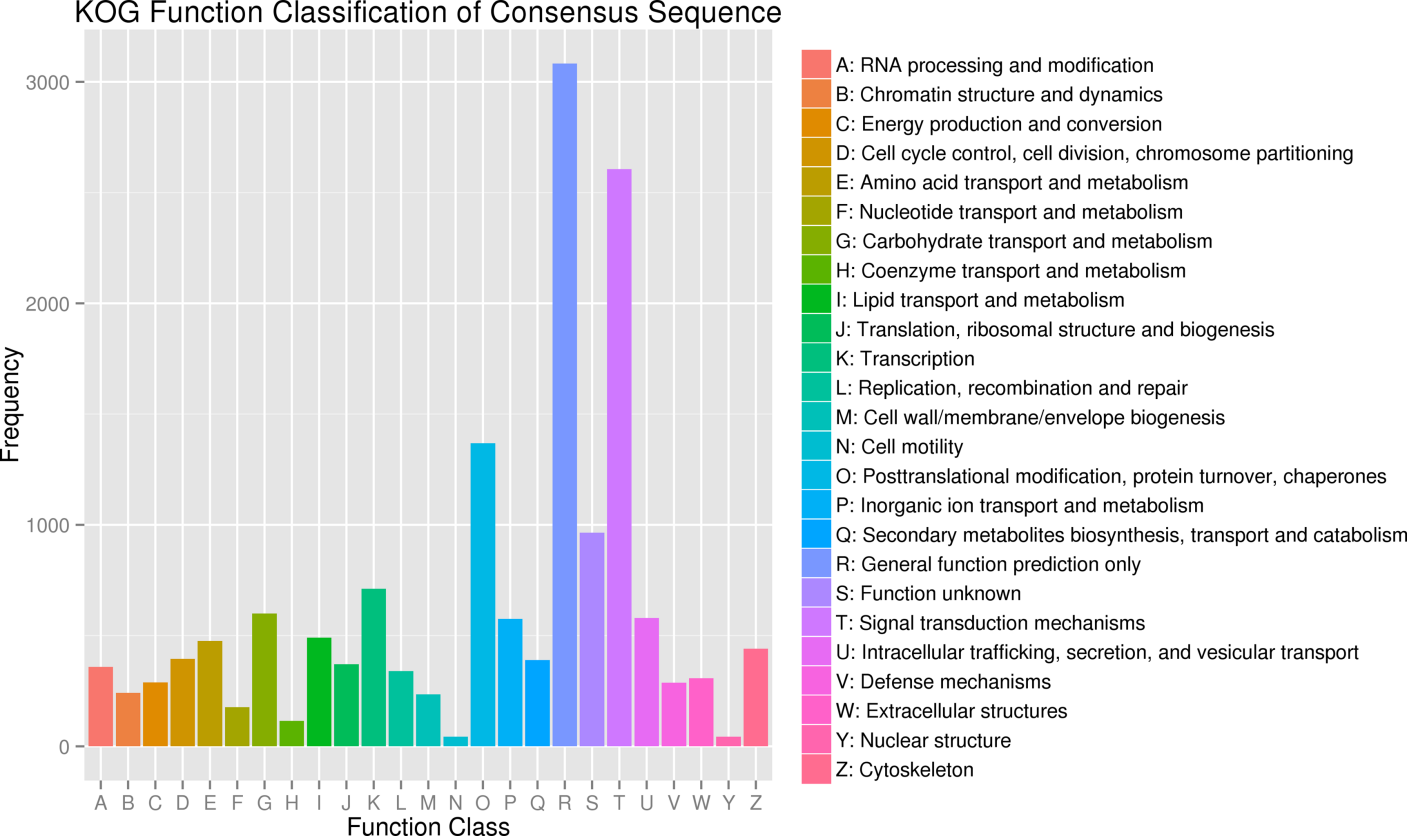
Eukaryotic Orthologous Groups (KOG) classification of the predicted genes. Results are summarized in 24 function classes according to their functions. The horizontal axis represents each class, and the vertical axis represents the frequency of the classes.

The predicted genes were annotated by aligning them to the NCBI non-redundant protein (nr) [46], non-redundant nucleotide (nt) [46], Swissprot (Swissprot, RRID:SCR_002380) [47], TrEMBL (TrEMBL, RRID:SCR_002380) [47], KOG [48], and KEGG (KEGG, RRID:SCR_001120) [49] databases using BLAST v2.2.31 [50] with a maximal e-value of 1e−5_;_ by aligning to the Pfam database (Pfam, RRID:SCR_004726) [51] using HMMer V3.0 [52]. Gene Ontology (GO) terms (Gene Ontology, RRID:SCR_002811) [53] were assigned to the genes using the BLAST2GO v2.5 pipeline (Blast2GO, RRID:SCR_005828) [54]. As a result, a total of 22,267 genes were annotated in ≥1 database (Table 3, Supplementary Table S12). Among the 21,897 genes annotated in the nr database, 11,772 genes (53.7%) showed homology with *C. gigas* hits (Supplementary Fig. S1). A total of 5,766 and 13,626 genes were annotated in the GO and KOG databases, respectively, and the functional classifications of these genes are presented in Figs 3 and 4, while the complete gene annotation table is reported in Supplementary Table 12.

**Table 3:** Statistics of gene annotation to different databases

Annotation database	Annotated number	Percentage (%)
GO_Annotation	5,766	23.98
KEGG_Annotation	9,174	38.15
KOG_Annotation	13,626	56.67
Pfam_Annotation	17,321	72.04
Swissprot_Annotation	12,866	53.51
TrEMBL_Annotation	21,887	91.03
nr_Annotation	21,897	91.07
nt_Annotation	12,786	53.18
All_Annotated	22,267	92.61

Finally, we predicted the non-coding RNAs based on the Rfam v12.1 (Rfam, RRID:SCR_007891) [55] and miRBase v21.0 (miRBase, RRID:SCR_003152) [56] databases. Putative microRNAs (miRNAs) and ribosomal RNAs (rRNAs) were predicted using Infernal v1.1 [57], and transnfer RNAs (tRNAs) were predicted with tRNAscan-SE v1.3.1 (tRNAscan-SE, RRID:SCR_010835) [58]. A total of 27 miRNAs, 204 rRNAs, and 1561 tRNAs were detected, corresponding to 15, 4, and 25 families, respectively.

## Availability of supporting data and materials

The DNA sequencing data and genome assembly have been deposited at the NCBI SRA database under the BioProject accession number PRJNA521075. Supporting data are also available via the *GigaScience* database GigaDB [59], and supporting protocols are archived in protocols.io [9].

## Additional files

**Supplementary Table S1:** Key protocols for chromosome-level genome assembly of *Scapharca* (*Anadara*) *broughtonii*.

**Supplementary Table S2:** Summary of the Illumina sequencing reads used for genome survey, correction, and evaluation.

**Supplementary Table S3:** Statistics of the length distribution of Pacbio subreads.

**Supplementary Table S4:** Statistics of the length distribution of Oxford Nanopore reads.

**Supplementary Table S5:** Statistics of the initial genome assembly of *Scapharca* (*Anadara*) *broughtonii*.

**Supplementary Table S6:** Summary of BUSCO analysis results.

**Supplementary Table S7:** Statistics of the mapping results of Hi-C reads.

**Supplementary Table S8:** Statistics of different types of the Hi-C reads.

**Supplementary Table S9:** Summary of the Hi-C assembly.

**Supplementary Table S10:** Statistics of the repeated sequences.

**Supplementary Table S11:** Summary of the gene prediction results.

**Supplementary Figure S1:** Species distribution of BLAST hits of the predicted genes in the nr database.

**Supplementary Table S12:** Integrated lists of gene annotation for the assembled *Scapharca* (*Anadara*) *broughtonii* genome.

giz067_GIGA-D-19-00008_Original_Submission

giz067_GIGA-D-19-00008_Revision_1

giz067_Response_to_Reviewer_Comments_Original_Submission

giz067_Reviewer_1_Report_Original_Submission Andrew Severin -- 3/15/2019 Reviewed

giz067_Reviewer_2_Report_Original_Submission Kevin Kocot -- 3/16/2019 Reviewed

giz067_Reviewer_2_Report_Revision_1 Kevin Kocot -- 4/30/2019 Reviewed

giz067_Supplemental_Files

## Abbreviations

BLAST: Basic Local Alignment Search Tool; bp: base pair; BUSCO: Benchmarking Universal Single-Copy Orthologs; EVM: EVidenceModeler; Gb: gigabase pair; GO: Gene Ontology; Hi-C: high-throughput chromosome conformation capture; kb: kilobase pair; KEGG: Kyoto Encyclopedia of Genes and Genomes; KOG: eukaryotic orthologous groups of proteins; Mb: megabase pair; miRNA: microRNA; NCBI: National Center for Biotechnology Information; nr: non-redundant protein; nt: non-redundant nucleotide; PacBio: Pacific Biosciences; PASA: Program to Assemble Spliced Alignments; PE: paired-end; p/n: part number; RNA-seq: RNA sequencing; rRNA: ribosomal RNA; SMRT: single-molecule real-time; tRNA: transfer RNA.

## Competing interests

The authors declare that they have no competing interests.

## Funding

This work was financially supported by National Key R&D Program of China (2018YFD0900304), China Agriculture Research System, grant number CARS-49, National Natural Science Foundation of China (31602142 and 31502208).

## Author’s Contributions

C.W., C.B., and Q.W. conceived the project; C.W., C.B., and Q.W. collected the samples; C.B., L.X., and Q.W. extracted the genomic DNA and performed genome sequencing; C.B., L.X., X.D., and U.R. analyzed the data; U.R., B.W., and Z.L. participated in discussions and provided valuable advice; C.B., L.X., U.R., B.W., and Z.L. wrote and revised the manuscript.

## References

[1] An HY, Park JY Ten new highly polymorphic microsatellite loci in the blood clam *Scapharca broughtoni*i. Mol Ecol Notes. 2005;5(4):896–8.

[2] Nishida K, Ishimura T, Suzuki A, et al. Seasonal changes in the shell microstructure of the bloody clam, *Scapharca broughtonii* (Mollusca: Bivalvia: Arcidae). Palaeogeogr Palaeocl. 2012;363:99–108.

[3] Boyd SE Order Arcoida. In: Beesley PL, Ross GJB, Wells A, eds. Mollusca: The Southern Synthesis Fauna of Australia, vol 5 Melbourne: CSIRO; 1998:253–61.

[4] Sugiura D, Katayama S, Sasa S, et al. Age and growth of the ark shell *Scapharca broughtonii* (Bivalvia, Arcidae) in Japanese waters. J Shellfish Res. 2014;33(1):315–24.

[5] Tang Q, Qiu XY, Wang J, et al. Resource enhancement of arkshell (*Scapharca* ( *Anadara*) *broughtonii*) in Shandong offshore waters. Chin J Appl Ecol. 1994;5(4):396–402.

[6] Bai C, Gao W, Wang C, et al. Identification and characterization of *Ostreid herpesvirus 1* associated with massive mortalities of *Scapharca broughtonii* broodstocks in China. Dis Aquat Organ. 2016;118(1):65–75. 26865236 10.3354/dao02958

[7] Zhao Q, Wu B, Liu ZH, et al. Molecular cloning, expression and biochemical characterization of hemoglobin gene from ark shell *Scapharca broughtonii*. Fish Shellfish Immunol. 2018;78:60–8. 29649584 10.1016/j.fsi.2018.03.038

[8] Bai CM, Xin LS, Rosani U, et al. Extraction of *Scapharca broughtonii* genomic DNA. protocols.io. 2019; 10.17504/protocols.io.zhaf32e.

[9] Bai CM, Xin LS, Rosani U, et al., Key protocols for chromosome-level genome assembly of the *Scapharca (Anadara) broughtonii*, protocols.io. 2019; 10.17504/protocols.io.zimf4c6.

[10] Koren S, Walenz BP, Berlin K, et al. Canu: scalable and accurate long-read assembly via adaptive k-mer weighting and repeat separation. Genome Res. 2017;27(5):722–36. 28298431 10.1101/gr.215087.116 PMC5411767

[11] Jayakumar V, Sakakibara Y Comprehensive evaluation of non-hybrid genome assembly tools for third-generation PacBio long-read sequence data. Brief Bioinform. 2017, doi:10.1093/bib/bbx147. PMC6585154 29112696

[12] Chakraborty M, Baldwin-Brown JG, Long AD, et al. Contiguous and accurate de novo assembly of metazoan genomes with modest long read coverage. Nucleic Acids Res. 2016;44(19):e147. 27458204 10.1093/nar/gkw654 PMC5100563

[13] Kurtz S, Phillippy A, Delcher AL, et al. Versatile and open software for comparing large genomes. Genome Biol. 2004;5(2):R12. 14759262 10.1186/gb-2004-5-2-r12 PMC395750

[14] Walker BJ, Abeel T, Shea T, et al. Pilon: an integrated tool for comprehensive microbial variant detection and genome assembly improvement. PLoS One. 2014;9(11):e112963. 25409509 10.1371/journal.pone.0112963 PMC4237348

[15] Bai CM, Xin LS, Rosani U, et al. The pipeline of assembly and annotation of the *Scapharca broughtonii* genome. protocols.io. 2019; 10.17504/protocols.io.z7zf9p6.

[16] Li H, Handsaker B, Wysoker A, et al. The Sequence Alignment/Map format and SAMtools. Bioinformatics. 2009;25(16):2078–9. 19505943 10.1093/bioinformatics/btp352 PMC2723002

[17] Simao FA, Waterhouse RM, Ioannidis P, et al. BUSCO: assessing genome assembly and annotation completeness with single-copy orthologs. Bioinformatics. 2015;31(19):3210–2. 26059717 10.1093/bioinformatics/btv351

[18] Li H Aligning sequence reads, clone sequences and assembly contigs with BWA-MEM. arXiv. 2013; https://arxiv.org/abs/1303.3997.

[19] Servant N, Varoquaux N, Lajoie BR, et al. HiC-Pro: an optimized and flexible pipeline for Hi-C data processing. Genome Biol. 2015;16:259. 26619908 10.1186/s13059-015-0831-x PMC4665391

[20] Bai CM, Xin LS, Rosani U, et al. The pipeline of Hi-C assembly of the *Scapharca broughtonii* genome. protocols.io. 2019; 10.17504/protocols.io.z8cf9sw.

[21] Burton JN, Adey A, Patwardhan RP, et al. Chromosome-scale scaffolding of de novo genome assemblies based on chromatin interactions. Nat Biotechnol. 2013;31(12):1119–25. 24185095 10.1038/nbt.2727 PMC4117202

[22] Ghurye J, Pop M, Koren S, et al. Scaffolding of long read assemblies using long range contact information. BMC Genomics. 2017;18:527. 28701198 10.1186/s12864-017-3879-z PMC5508778

[23] Zhou L, Wang ZC Studies on karyotype analysis in the *Scapharca broughtonii*. J Fish Chin. 1997;21(4):455–7.

[24] Xu Z, Wang H LTR_FINDER: an efficient tool for the prediction of full-length LTR retrotransposons. Nucleic Acids Res. 2007;35(Web Server issue):W265–8. 17485477 10.1093/nar/gkm286 PMC1933203

[25] Price AL, Jones NC, Pevzner PA De novo identification of repeat families in large genomes. Bioinformatics. 2005;21(Suppl 1):i351–8. 15961478 10.1093/bioinformatics/bti1018

[26] Edgar RC, Myers EW PILER: identification and classification of genomic repeats. Bioinformatics. 2005;21(Suppl 1):i152–8. 15961452 10.1093/bioinformatics/bti1003

[27] Wicker T, Sabot F, Hua-Van A, et al. A unified classification system for eukaryotic transposable elements. Nat Rev Genet. 2007;8(12):973–82. 17984973 10.1038/nrg2165

[28] Jurka J, Kapitonov VV, Pavlicek A, et al. Repbase Update, a database of eukaryotic repetitive elements. Cytogenet Genome Res. 2005;110(1–4):462–7. 16093699 10.1159/000084979

[29] Tarailo-Graovac M, Chen N Using RepeatMasker to identify repetitive elements in genomic sequences. Curr Protoc Bioinformatics. 2009;Chapter 4:Unit 4 10, doi:10.1002/0471250953.bi0410s25. 19274634

[30] Burge C, Karlin S Prediction of complete gene structures in human genomic DNA. J Mol Biol. 1997;268(1):78–94. 9149143 10.1006/jmbi.1997.0951

[31] Stanke M, Waack S Gene prediction with a hidden Markov model and a new intron submodel. Bioinformatics. 2003;19(Suppl 2):ii215–25. 14534192 10.1093/bioinformatics/btg1080

[32] Majoros WH, Pertea M, Salzberg SL TigrScan and GlimmerHMM: two open source ab initio eukaryotic gene-finders. Bioinformatics. 2004;20(16):2878–9. 15145805 10.1093/bioinformatics/bth315

[33] Blanco E, Parra G, Guigó R Using geneid to identify genes. Curr Protoc Bioinformatics. 2007;18(1):4.3.1–4.3.28. 10.1002/0471250953.bi0403s18 18428791

[34] Korf I Gene finding in novel genomes. BMC Bioinformatics. 2004;5:59. 15144565 10.1186/1471-2105-5-59 PMC421630

[35] Keilwagen J, Wenk M, Erickson JL, et al. Using intron position conservation for homology-based gene prediction. Nucleic Acids Res. 2016;44(9):e89. 26893356 10.1093/nar/gkw092 PMC4872089

[36] Bai CM, Rosani U, Xin LS, et al. Dual transcriptomic analysis of *Ostreid herpesvirus 1* infected *Scapharca broughtonii* with an emphasis on viral anti-apoptosis activities and host oxidative bursts. Fish Shellfish Immun. 2018;82:554–64. 10.1016/j.fsi.2018.08.054 30165154

[37] Campbell MA, Haas BJ, Hamilton JP, et al. Comprehensive analysis of alternative splicing in rice and comparative analyses with *Arabidopsis*. BMC Genomics. 2006;7:327. 17194304 10.1186/1471-2164-7-327 PMC1769492

[38] Pertea M, Kim D, Pertea GM, et al. Transcript-level expression analysis of RNA-seq experiments with HISAT, StringTie and Ballgown. Nat Protoc. 2016;11(9):1650–67. 27560171 10.1038/nprot.2016.095 PMC5032908

[39] Haas BJ, Papanicolaou A. TransDecoder (Find Coding Regions Within Transcripts). http://transdecoder.github.io. Accessed on 13 Feb 2017.

[40] Tang S, Lomsadze A, Borodovsky M Identification of protein coding regions in RNA transcripts. Nucleic Acids Res. 2015;43(12):e78. 25870408 10.1093/nar/gkv227 PMC4499116

[41] Haas BJ, Salzberg SL, Zhu W, et al. Automated eukaryotic gene structure annotation using EVidenceModeler and the Program to Assemble Spliced Alignments. Genome Biol. 2008;9(1):R7. 18190707 10.1186/gb-2008-9-1-r7 PMC2395244

[42] Xiao J, Sekhwal MK, Li P, et al. Pseudogenes and their genome-wide prediction in plants. Int J Mol Sci. 2016;17(12):1991. 10.3390/ijms17121991 PMC5187791 27916797

[43] Thibaud-Nissen F, Ouyang S, Buell CR Identification and characterization of pseudogenes in the rice gene complement. BMC Genomics. 2009;10:317. 19607679 10.1186/1471-2164-10-317 PMC2724416

[44] She R, Chu SC, Uyar B, et al. genBlastG: using BLAST searches to build homologous gene models. Bioinformatics. 2011;27(15):2141–3. 21653517 10.1093/bioinformatics/btr342

[45] Birney E, Clamp M, Durbin R GeneWise and Genomewise. Genome Res. 2004;14(5):988–95. 15123596 10.1101/gr.1865504 PMC479130

[46] Marchler-Bauer A, Lu S, Anderson JB, et al. CDD: a Conserved Domain Database for the functional annotation of proteins. Nucleic Acids Res. 2011;39(Database issue):D225–9. 21109532 10.1093/nar/gkq1189 PMC3013737

[47] Boeckmann B, Bairoch A, Apweiler R, et al. The SWISS-PROT protein knowledgebase and its supplement TrEMBL in 2003. Nucleic Acids Res. 2003;31(1):365–70. 12520024 10.1093/nar/gkg095 PMC165542

[48] Tatusov RL, Natale DA, Garkavtsev IV, et al. The COG database: new developments in phylogenetic classification of proteins from complete genomes. Nucleic Acids Res. 2001;29(1):22–8. 11125040 10.1093/nar/29.1.22 PMC29819

[49] Kanehisa M, Goto S KEGG: Kyoto Encyclopedia of Genes and Genomes. Nucleic Acids Res. 2000;28(1):27–30. 10592173 10.1093/nar/28.1.27 PMC102409

[50] Altschul SF, Gish W, Miller W, et al. Basic Local Alignment Search Tool. J Mol Biol. 1990;215(3):403–10. 2231712 10.1016/S0022-2836(05)80360-2

[51] El-Gebali S, Mistry J, Bateman A, et al. The Pfam protein families database in 2019. Nucleic Acids Res. 2019;47(D1):D427–32. 30357350 10.1093/nar/gky995 PMC6324024

[52] Eddy SR, Mitchison G, Durbin R Maximum discrimination hidden Markov models of sequence consensus. J Comput Biol. 1995;2(1):9–23. 7497123 10.1089/cmb.1995.2.9

[53] Dimmer EC, Huntley RP, Alam-Faruque Y, et al. The UniProt-GO Annotation database in 2011. Nucleic Acids Res. 2012;40(Database issue):D565–70. 22123736 10.1093/nar/gkr1048 PMC3245010

[54] Conesa A, Gotz S, Garcia-Gomez JM, et al. Blast2GO: a universal tool for annotation, visualization and analysis in functional genomics research. Bioinformatics. 2005;21(18):3674–6. 16081474 10.1093/bioinformatics/bti610

[55] Griffiths-Jones S, Moxon S, Marshall M, et al. Rfam: annotating non-coding RNAs in complete genomes. Nucleic Acids Res. 2005;33(Database issue):D121–4. 15608160 10.1093/nar/gki081 PMC540035

[56] Griffiths-Jones S, Grocock RJ, van Dongen S, et al. miRBase: microRNA sequences, targets and gene nomenclature. Nucleic Acids Res. 2006;34(Database issue):D140–4. 16381832 10.1093/nar/gkj112 PMC1347474

[57] Nawrocki EP, Eddy SR Infernal 1.1: 100-fold faster RNA homology searches. Bioinformatics. 2013;29(22):2933–5. 24008419 10.1093/bioinformatics/btt509 PMC3810854

[58] Lowe TM, Eddy SR tRNAscan-SE: a program for improved detection of transfer RNA genes in genomic sequence. Nucleic Acids Res. 1997;25(5):955–64. 9023104 10.1093/nar/25.5.955 PMC146525

[59] Bai C, Xin LS, Rosani U, et al. Supporting data for “Chromosomal-level assembly of the blood clam, *Scapharca (Anadara) broughtonii*, using long sequence reads and Hi-C.”. GigaScience Database. 2019 10.5524/100607. PMC6615981 31289832

