# Supplementary material for: Chromosomal-level assembly of the blood clam, Scapharca (Anadara) broughtonii, using long sequence reads and Hi-C
Source: Gigascience. 2019 Jul 9;8(7):giz067. doi: 10.1093/gigascience/giz067 (PMC6615981; doi:10.1093/gigascience/giz067)
Supplement: giz067_GIGA-D-19-00008_Original_Submission [file giz067_giga-d-19-00008_original_submission.pdf]

GigaScience

Chromosomal-level assembly of the bloody clam, Scapharca (Anadara) broughtonii, using long sequence reads and Hi-C

--Manuscript Draft--

<table>
<tr><td>Manuscript Number:</td><td>GIGA-D-19-00008</td></tr>
<tr><td>Full Title:</td><td>Chromosomal-level assembly of the bloody clam, Scapharca (Anadara) broughtonii, using long sequence reads and Hi-C</td></tr>
<tr><td>Article Type:</td><td>Data Note</td></tr>
<tr><td>Funding Information:</td><td><table>
<tr><td>National Key R&D Program of China
(2018YFD0900304)</td><td>Dr. Biao Wu</td></tr>
<tr><td>China Agriculture Research System
(CARS-49)</td><td>Prof. Chong-Ming Wang</td></tr>
<tr><td>National Natural Science Foundation of China
(31602142)</td><td>Dr. Biao Wu</td></tr>
<tr><td>National Natural Science Foundation of China
(31502208)</td><td>Dr. Chang-Ming Bai</td></tr>
</table></td></tr>
<tr><td>Abstract:</td><td>Background: The bloody clam, Scapharca (Anadara) broughtonii, is an economically and ecologically important marine bivalve of the Family Arcidae. Many efforts have been made to study their population genetics, breeding, cultivation and stock enrichment. However, the lack of a reference genome has hindered these researches. Here, we reported the complete genome sequence of S. broughtonii, a first reference genome of the Family Arcidae.
Funding: A total of 75.79 Gb clean data of long reads was generated with the PacBio and Oxford Nanopore platforms, which represented approx. 86× coverage of the bloody clam genome. De novo assembly of the long reads generated an 884.5 Mb genome of the bloody clam with a contig N50 of 1.80 Mb and scaffold N50 of 45.00 Mb, respectively. Hi-C scaffolding of the genome resulted in 19 chromosomes containing 99.35% bases of the assembled genome. Genome annotation revealed that a considerable part of the genome (46.1%) is composed by repeated sequences. Gene prediction identified 24,045 protein-coding genes, and 84.7% of them were annotated in at least one database.
Conclusion: We report here the chromosomal-level assembly of the bloody clam with long sequence reads and Hi-C scaffolding. The genomic data could be served as reference genome and provide a valuable resource for various studies related to genomic information of bloody clam.</td></tr>
<tr><td>Corresponding Author:</td><td>Chong-Ming Wang
YSFRI
Qingdao, Shandong CHINA</td></tr>
<tr><td>Corresponding Author Secondary Information:</td><td></td></tr>
<tr><td>Corresponding Author's Institution:</td><td>YSFRI</td></tr>
<tr><td>Corresponding Author's Secondary Institution:</td><td></td></tr>
<tr><td>First Author:</td><td>Chang-Ming Bai, Ph.D.</td></tr>
<tr><td>First Author Secondary Information:</td><td></td></tr>
<tr><td>Order of Authors:</td><td>Chang-Ming Bai, Ph.D.</td></tr>
<tr><td></td><td>Lu-Sheng Xin</td></tr>
<tr><td></td><td>Umberto Rosani</td></tr>
<tr><td></td><td>Biao Wu</td></tr>
<tr><td></td><td>Qing-Chen Wang</td></tr>
<tr><td></td><td></td></tr>
</table>

Powered by Editorial Manager® and ProduXion Manager® from Aries Systems Corporation

	Xiao-Ke Duan
	Zhi-Hong Liu
	Chong-Ming Wang
**Order of Authors Secondary Information:**	
**Additional Information:**	
**Question**	**Response**
Are you submitting this manuscript to a special series or article collection?	No
**Experimental design and statistics**<br><br>Full details of the experimental design and statistical methods used should be given in the Methods section, as detailed in our Minimum Standards Reporting Checklist. Information essential to interpreting the data presented should be made available in the figure legends.<br><br>Have you included all the information requested in your manuscript?	Yes
**Resources**<br><br>A description of all resources used, including antibodies, cell lines, animals and software tools, with enough information to allow them to be uniquely identified, should be included in the Methods section. Authors are strongly encouraged to cite Research Resource Identifiers (RRIDs) for antibodies, model organisms and tools, where possible.<br><br>Have you included the information requested as detailed in our Minimum Standards Reporting Checklist?	Yes
**Availability of data and materials**<br><br>All datasets and code on which the conclusions of the paper rely must be either included in your submission or deposited in publicly available repositories (where available and ethically	Yes

appropriate), referencing such data using a unique identifier in the references and in the “Availability of Data and Materials” section of your manuscript.<br><br>Have you have met the above requirement as detailed in our Minimum Standards Reporting Checklist?	

Powered by Editorial Manager® and ProduXion Manager® from Aries Systems Corporation

Manuscript

Click here to access/download;Manuscript;ArkshellGenome_12Feb_UR.docx

Click here to view linked References

Chromosomal-level assembly of the bloody clam, *Scapharca* (*Anadara*) *broughtonii*, using long sequence reads and Hi-C

Chang-Ming Bai[a]†, Lu-Sheng Xin[a]†, Umberto Rosani[b], Biao Wu[a], Qing-Chen Wang[a], Xiao-Ke Duan[c], Zhi-Hong Liu[a], Chong-Ming Wang[a]*

[a] *Key Laboratory of Maricultural Organism Disease Control, Ministry of Agriculture; Laboratory for Marine Fisheries Science and Food Production Processes, Qingdao National Laboratory for Marine Science and Technology; Qingdao Key Laboratory of Mariculture Epidemiology and Biosecurity; Yellow Sea Fisheries Research Institute, Chinese Academy of Fishery Sciences, Qingdao 266071, China*

[b] *Department of Biology, University of Padua, Padua 35121, Italy*

[c] *Biomarker Technologies Corporation, Beijing 101200, China*

† These authors contributed equally to this work.

* Correspondence to: Chong-Ming Wang, E-mail address: wangcm@ysfri.ac.cn.

Abstract

Background: The bloody clam, *Scapharca* (*Anadara*) *broughtonii*, is an economically and ecologically important marine bivalve of the Family Arcidae. Many efforts have been made to study their population genetics, breeding, cultivation and stock enrichment. However, the lack of a reference genome has hindered these researches. Here, we reported the complete genome sequence of *S. broughtonii,* a first reference genome of the Family Arcidae.

Funding: A total of 75.79 Gb clean data of long reads was generated with the PacBio and Oxford Nanopore platforms, which represented approx. 86× coverage of the bloody clam genome. *De novo* assembly of the long reads generated an 884.5 Mb genome of the bloody clam with a contig N50 of 1.80 Mb and scaffold N50 of 45.00 Mb, respectively. Hi-C scaffolding of the genome resulted in 19 chromosomes containing 99.35% bases of the assembled genome. Genome annotation revealed that a considerable part of the genome (46.1%) is composed by repeated sequences. Gene prediction identified 24,045 protein-coding genes, and 84.7% of them were annotated in at least one database.

Conclusion: We report here the chromosomal-level assembly of the bloody clam with long sequence reads and Hi-C scaffolding. The genomic data could be served as reference genome and provide a valuable resource for various studies related to genomic information of bloody clam.

Keywords: bloody calm; PacBio; Hi-C; genomic; chromosomal assembly.

Background information

The bloody clam, *Scapharca* (*Anadara*) *broughtonii* (Schrenck, 1867), also known as ark shell, belongs to the Family Arcidae, Class Pteriomorphia, Phylum Mollusca. Approx. 200 species are found in this family, most of them distributed in tropical areas [1]. Differently, the bloody clam lived in temperate areas along the coasts of northern China, Japan, Korea and the Russian Far East [1, 2]. The name "bloody clam" originated from the red color of their visceral mass due to the presence of hemoglobin in both tissues and hemolymph [1, 2]. Containing hemoglobin is not typical of mollusk, and one of the most interesting points of Family Arcidae. Bloody clam has thick and harder calcareous shells and is relatively large in size, which could grow to 100 mm in shell length [3]. The shells are always covered by hairy periostracum colored in brown [2]. Served as a source of sashimi, the wild bloody clam resource had been overused to depletion in the last century. Many efforts have been made to recover the wild population of bloody clam in China, Japan and Korea. Many research and production process involved the cultivation of them in high density, and rendered them to pathogenic bacterial and virus [1, 4-6]. Compared to oysters and scallops, we still knew very little about the basic biology and cultivation of bloody clam and little information is available regarding the genomic sequence of the bloody clam. Here, we sequenced the complete genome of the bloody clam to provide a genomic foundation for future research and culture industry development.

Sample collection and sequencing

To overcome the excessive polysaccharide content of bloody clam tissues, we extracted high-quality genomic DNA from haemocytes, which were collected from a batch of adults sampled from wild populations near Jimo, Shandong Province, China. The DNA was extracted using DNeasy® Blood & Tissue Kit (QIAGEN, Cat No.: 69504) with slight modification to remove polysaccharide. The DNA quality and quantity were measured with agarose gel electrophoresis and Qubit 3.0 (Invitrogen, Carlsbad, CA, USA), respectively. High-quality DNA was sent to BioMarker Technology Co. Ltd. (Beijing, China) for libraries preparation and high-throughput sequencing using PacBio, Nanopore and Illumina platforms (Table 1).

PacBio sequencing was carried out with the SMRT Bell™ library using a DNA Template Prep Kit 1.0 (PacBio p/n 100-259-100). Briefly, the genomic DNA (10 μg) was mechanically sheared using a Covaris g-Tube (Kbiosciences p/n 520079) to get DNA fragments of approx. 20 Kb in size. The

sheared DNA was DNA-damage repaired and end-repaired using polishing enzymes. Then a blunt-end ligation reaction followed by exonuclease treatment was conducted to generate the SMRT Bell™ template. Finally, large fragments (>10 Kb) were enriched with Blue Pippin device (Sage Science, Inc., Beverly, MA, USA) for sequencing. A total of 15 SMRT cells were processed, of which 7 and 8 cells were sequenced with Sequel and RS II instruments (Pacific Biosciences, Menlo Park, CA, USA), respectively. A total of 67.32 Gb PacBio data was generated. For Oxford Nanopore sequencing, approx. 5 μg genomic DNA was sheared and size-selected (~20 kb) with the same procedure as described above. The selected fragments were further processed using the Ligation Sequencing 1D Kit (Oxford Nanopore, Oxford, UK) according to the manufacturer's instructions, and sequenced using the MinION portable DNA sequencer with the 48 hours run script (Oxford Nanopore) for a total of 8.47 Gb data. For Illumina sequencing, paired-end (PE) libraries with insert size of 350 bp were constructed according to the manufacturer's protocol and sequenced with an Illumina HiSeq X Ten platform (San Diego, CA, USA) with paired-end 150 (PE150) strategy. A total of 53.06 Gb Illumina data was generated and used for genome survey, correction and evaluation (Supplementary Table S1). All of the long-reads data for assembly and Illumina data for genome survey were deposited in the NCBI SRA database under the SAMN10879241.

Initial genome assembly and evaluation

The Sequel raw bam and RS II H5 files were converted into subreads in fasta format with the standard PacBio SMRT software package. Consequently, a total of 63,330,577,481 and 3,990,849,516 bases were obtained with Sequel and RS II instruments, respectively. After subreads shorter than 500 bp in size were filtered out, we obtained a clean dataset of 4,761,097 reads with a total of 67,260,156,459 bases (Supplementary Table S2). The N50 and mean length of these subreads were 21,932 and 14,127 bp, respectively. The Nanopore raw reads were base-called from their raw FAST5 files using Guppy implanted in MinKNOW (Oxford Nanopore, Oxford, UK). Applying a minimum length cutoff of 500 bp, we produced a total of 8,468,912,896 bases data (Supplementary Table S3). Hybrid assembly of all of the filtered reads were carried out using Canu (v1.5) [7] and WTDBG (v1.2.8) [8] tools and the two assemblies were joined with Quickmerge [9], removing the redundancy with Numer [10]. Finally, the genome assembly was corrected using the Illumina reads using Pilon v1.22 (Pilon, RRID: SCR 014731) with default settings [11]. The initial genome assembly was

884,500,940 bp in length with a contig N50 of 2,388,811 bp (Supplementary Table 4).

To evaluate the genome assembly, the assembled genome was firstly subjected to aligning with the 360,937,442 Illumina reads generated in the present study with SAMTools (SAMTOOLS, RRID:SCR_002105) [12], and then subjected to comparison with 303 conservative genes in eukaryote and 978 genes in metazoan with BUSCO v2.0 (BUSCO, RRID:SCR 015008) [13], respectively. As a result, 97.45 % of the Illumina reads were successfully mapped to the assembled genome. The BUSCO analysis found 273 and 897 conservative genes belonging to eukaryote and metazoan datasets, accounting for 90.10% and 91.72% of the totals, respectively (Supplementary Table 5). Thus, the high alignment ratios revealed in the two above analysis demonstrated the high quality of contig assembly for the bloody clam.

Hi-C analysis and chromosome assembly

For the Hi-C library, fresh adductor muscle was fixed using formaldehyde with a final concentration of 1%. The fixed DNA was then digested with the restriction enzyme (*Hind* III), followed by 5′ repairing and labeling with a biotinylated residue. Subsequently, the digested and labeled DNA was ligated, reversed and sheared to a length of 300-700 bp and purified as previously described [14]. Finally, the purified fragments were used for library preparation as described above and sequenced using an Illumina HiSeq X Ten platform with 150 paired-end mode. A total of 174,148,156 read pairs (52.16 Gb) with a Q30 of 93.16% were generated and used for the Hi-C analysis (NCBI SRA accession number: SAMN10879242).

To get the unique mapped read pairs, the 174 million read pairs were first truncated at the putative Hi-C junctions and then the resulting trimmed reads were aligned to the assembly results using BWA aligner (BWA, RRID:SCR_010910) and applying default parameters [15]. Only uniquely aligned pairs whose mapping qualities higher than 20 were considered for further analysis. A total of 206 million reads (59.23%) were mapped to the assembled genome, of which 51 million read pairs (29.33%) were unique mapped read pairs (Supplementary Table 6). Then, the invalid interaction pairs due to self-circle ligation, dangling ends, re-ligation and the other dumped types were filtered out with HiC-Prov2.8.1 [16]. After filtration, we obtained 17 million valid interaction pairs (Supplementary Table 7), accounting for 33.66% of the unique mapped read pairs, which were used for the Hi-C analysis.

For chromosome assembly, the pre-assembled contigs were broken into equal length of 300bp and reassembled with the agglomerative hierarchical clustering method implanted in Lachesis [17]. Finally, 1384 contigs (82.53%) were successfully clustered into 19 groups (Figure 1), which was consistent with the previous karyotype analyses of the bloody clam [18]. The 1384 clustered contigs correspond to 878.79 Mb in length, accounting for 99.35% of the total length of the assembled genome. Further analysis with Lachesis showed that 670 contigs corresponding to 819.17 Mb were anchored with defined order and orient, accounting for 48.41% and 93.22% of the total genome by contig number and length, respectively (Supplementary Table 8). Finally, we obtained a chromosomal-level bloody clam assembly with a contig N50 of 1.80 Mb and scaffold N50 of 45.00 Mb, which represented the first reference genome of Family Arcidae (Table 2).

Genome annotation

We used LTR FINDER v1.05 (LTR_Finder, RRID:SCR_015247) [19], RepeatScout v1.0.5 (RepeatScout, RRID:SCR 014653) [20] and PILER-DF v2.4 [21] to construct a repetitive sequence library based on bloody clam genome. Then we used PASTEClassifier v1.0 [22] to classify these repeats and we merged them with the ones available in the Repbase database [23]. Finally, based on the constructed library, the repeat sequences of the assembled genome were identified with RepeatMasker v4.0.6 (RepeatMasker, RRID:SCR 012954) [24]. A total of 407.8 Mb sequence was identified as repeated sequence, representing 46.1% of the total genome length. The statistics of number, length and percentage of each repeat type could be found in supplementary table 9.

We then predicted the protein-coding genes using the following approaches: *ab initio* prediction, homology-based prediction, and transcriptome-based prediction. For *ab initio* prediction, Genscan v1.0 (Genscan, RRID:SCR 012902) [25], Augustus v2.4 (Augustus, RRID:SCR 008417) [26], GlimmerHMM v3.0.4 (GlimmerHMM, RRID:SCR 002654) [27], GeneID v1.4 [28] and SNAP v2006-07-28 (SNAP, RRID:SCR 002127) [29] were used. For homology-based prediction, protein sequences of three closely related mollusk species (*Crassostrea gigas, Mizuhopecten yessoensis* and *Mytilus galloprovincialis*) and *Danio rerio* were downloaded from NCBI (NCBI, RRID:SCR_006472) and aligned against the assembled genome with GeMoMa v1.3.1 [30]. For the transcriptome-based prediction, transcriptomic data obtained from a previous study (NCBI SRA accession ID: PRJNA450478) was used as input data [31]. This data have been *de novo* assembled with Trinity

software in the previous study [31] and the prediction was carried out with PASA v2.0.2 (PASA, RRID:SCR_014656) [32] based on the assembled unigenes. We also performed reference-based assembly of the RNA-seq data with Hisat v2.0.4 (HISAT2, RRID:SCR_015530) and Stringtie v1.2.3 [33], then predicted with TransDecoder v2.0 (http://transdecoder.github.io) and GeneMark v5.1 (GeneMark, RRID:SCR_011930) [34]. Finally, the results from the three approaches were integrated using EVM v1.1.1 (EVM, RRID:SCR 014659) [35] and polished with PASA v2.0.2. A total of 24,045 genes with an average length of 12,549 bp were predicted from the bloody clam genome assembly (Supplementary Table 10). Pseudogenes were predicted with GeneWise v2.4.1 (GeneWise, RRID:SCR 015054) [36], obtaining 1,658 pseudogenes with an average length of 3150.8 bp.

The predicted genes were annotated by aligning them to the NCBI non-redundant protein sequences (nr) [37], non-redundant nucleotide (nt) [37], Swissprot (Swissprot, RRID:SCR 002380) [38], TrEMBL (TrEMBL, RRID:SCR 002380) [38], KOG [39] and KEGG (KEGG, RRID:SCR 001120) [40] databases using the BLAST [41] with a maximal e-value of 1e^{-5}; by aligning to the Pfam database (Pfam, RRID:SCR 004726) [42] using hmmer V3.0 [43], by aligning to GO (Gene Ontology, RRID:SCR_002811) [44] terms using the BLAST2GO pipeline (Blast2GO, RRID:SCR_005828) [45]. As a result, a total of 22,308 genes were annotated to at least one database (Table 3, Supplementary 11). There were 21,897 genes annotated in nr database, of which 11,772 genes (53.7%) were homologous to *C. gigas* hits (Supplementary Figure 1). There were 5,766 and 13,626 genes annotated in GO and KOG databases respectively, and the functional classification of these genes were presented in Figure 2 and 3, respectively.

Finally, we predicted non-coding RNAs in the assembled genome of bloody clam based on Rfam (Rfam, RRID:SCR_007891) [46] and miRBase (miRBase, RRID:SCR_003152) [47] databases. miRNA and rRNA were predicted using Infenal 1.1 [48], tRNA was predicted with tRNAscan-SE v1.3.1 (tRNAscan-SE, RRID:SCR_010835) [49]. A total of 27 miRNAs, 204 rRNAs and 1561 tRNAs were detected, corresponding to 15, 4 and 25 families, respectively.

Additional files

Supplementary material.docx

Supplementary table11.xlsx

Availability of Data and Materials

The DNA sequencing data and genome assembly have been deposited in NCBI under the BioProject accession number PRJNA521075. Supporting data are also available via the GigaScience database GigaDB.

Abbreviations

BLAST: Basic Local Alignment Search Tool; bp: base pair; BUSCO: Benchmarking Universal Single-Copy Orthologs; Gb: gigabase; GO: Gene Ontology; Hi-C: high-throughput chromosome conformation capture; KEGG: Kyoto Encyclopedia of Genes and Genomes; KOG: eukaryotic orthologous groups of proteins; Mb: megabase; NCBI: National Center for Biotechnology Information; PacBio: Pacific Biosciences; RNA-seq: RNA sequencing; SMRT: single-molecule real-time.

Competing interests

The authors declare that they have no competing interests.

Funding

This work was financially supported by National Key R&D Program of China (2018YFD0900304), China Agriculture Research System, grant number CARS-49, National Natural Science Foundation of China (31602142 and 31502208).

Author Contributions: C.W., C.B. and Q.W. conceived the project; C.W., C.B. and Q.W. collected the samples; C.B., L.X. and Q.W. extracted the genomic DNA and performed genome sequencing; C.B., L.X. X.D. and U.R. analyzed the data; U.R., B.W. and Z.L. participated in discussions and provided valuable advice; C.B., L.X., U.R., B.W. and Z.L. wrote and revised the manuscript.

Reference:

1. An HY and Park JY. Ten new highly polymorphic microsatellite loci in the blood clam *Scapharca broughtonii*. Mol Ecol Notes. 2005;5 4:896-8. doi:DOI 10.1111/j.1471-8286.2005.01104.x.
2. Nishida K, Ishimura T, Suzuki A and Sasaki T. Seasonal changes in the shell microstructure of the bloody clam, *Scapharca broughtonii* (Mollusca: Bivalvia: Arcidae). Palaeogeogr Palaeocl. 2012;363:99-108. doi:10.1016/j.palaeo.2012.08.017.
3. Sugiura D, Katayama S, Sasa S and Sasaki K. Age And Growth Of the Ark Shell *Scapharca Broughtonii* (Bivalvia, Arcidae) In Japanese Waters. J Shellfish Res. 2014;33 1:315-24. doi:10.2983/035.033.0130.
4. Tang Q, Qiu X, Wang J, Guo X and Yang A. Resource enhancement of arkshell (*Scapharca* (*Anadara*) *broughtonii*) in Shandong offshore waters. Chinese Journal of Applied Ecology. 1994;5 4:396-402.
5. Bai C, Gao W, Wang C, Yu T, Zhang T, Qiu Z, et al. Identification and characterization of ostreid herpesvirus 1 associated with massive mortalities of *Scapharca broughtonii* broodstocks in China. Dis Aquat Organ. 2016;118 1:65-75. doi:10.3354/dao02958.
6. Zhao Q, Wu B, Liu Z, Sun X, Zhou L, Yang A, et al. Molecular cloning, expression and biochemical characterization of hemoglobin gene from ark shell *Scapharca broughtonii*. Fish Shellfish Immunol. 2018;78:60-8. doi:10.1016/j.fsi.2018.03.038.
7. Koren S, Walenz BP, Berlin K, Miller JR, Bergman NH and Phillippy AM. Canu: scalable and accurate long-read assembly via adaptive k-mer weighting and repeat separation. Genome Res. 2017;27 5:722-36. doi:10.1101/gr.215087.116.
8. Jayakumar V and Sakakibara Y. Comprehensive evaluation of non-hybrid genome assembly tools for third-generation PacBio long-read sequence data. Brief Bioinform. 2017; doi:10.1093/bib/bbx147.
9. Chakraborty M, Baldwin-Brown JG, Long AD and Emerson JJ. Contiguous and accurate de novo assembly of metazoan genomes with modest long read coverage. Nucleic Acids Res. 2016;44 19:e147. doi:10.1093/nar/gkw654.
10. Kurtz S, Phillippy A, Delcher AL, Smoot M, Shumway M, Antonescu C, et al. Versatile and open software for comparing large genomes. Genome Biol. 2004;5 2:R12. doi:10.1186/gb-2004-5-2-r12.
11. Walker BJ, Abeel T, Shea T, Priest M, Abouelliel A, Sakthikumar S, et al. Pilon: an integrated tool for comprehensive microbial variant detection and genome assembly improvement. Plos One. 2014;9 11:e112963. doi:10.1371/journal.pone.0112963.
12. Li H, Handsaker B, Wysoker A, Fennell T, Ruan J, Homer N, et al. The Sequence Alignment/Map format and SAMtools. Bioinformatics. 2009;25 16:2078-9. doi:10.1093/bioinformatics/btp352.
13. Simao FA, Waterhouse RM, Ioannidis P, Kriventseva EV and Zdobnov EM. BUSCO: assessing genome assembly and annotation completeness with single-copy orthologs. Bioinformatics. 2015;31 19:3210-2. doi:10.1093/bioinformatics/btv351.
14. Rao SSP, Huntley MH, Durand NC, Stamenova EK, Bochkov ID, Robinson JT, et al. A 3D Map of the Human Genome at Kilobase Resolution Reveals Principles of Chromatin Looping. Cell. 2014;159 7:1665-80. doi:10.1016/j.cell.2014.11.021.

15. Li H. Aligning sequence reads, clone sequences and assembly contigs with BWA-MEM. arXiv. 2013;1303.3997.

16. Servant N, Varoquaux N, Lajoie BR, Viara E, Chen CJ, Vert JP, et al. HiC-Pro: an optimized and flexible pipeline for Hi-C data processing. Genome Biology. 2015;16 doi:10.1186/s13059-015-0831-x.

17. Burton JN, Adey A, Patwardhan RP, Qiu R, Kitzman JO and Shendure J. Chromosome-scale scaffolding of de novo genome assemblies based on chromatin interactions. Nat Biotechnol. 2013;31 12:1119-25. doi:10.1038/nbt.2727.

18. Zhou L and Wang Z-C. Studies on karyotype analysis in the *Scapharca broughtonii*. Journal of Fisheries of China. 1997;21 4:455-7.

19. Xu Z and Wang H. LTR_FINDER: an efficient tool for the prediction of full-length LTR retrotransposons. Nucleic Acids Res. 2007;35 Web Server issue:W265-8. doi:10.1093/nar/gkm286.

20. Price AL, Jones NC and Pevzner PA. De novo identification of repeat families in large genomes. Bioinformatics. 2005;21 Suppl 1:i351-8. doi:10.1093/bioinformatics/bti1018.

21. Edgar RC and Myers EW. PILER: identification and classification of genomic repeats. Bioinformatics. 2005;21 Suppl 1:i152-8. doi:10.1093/bioinformatics/bti1003.

22. Wicker T, Sabot F, Hua-Van A, Bennetzen JL, Capy P, Chalhoub B, et al. A unified classification system for eukaryotic transposable elements. Nat Rev Genet. 2007;8 12:973-82. doi:10.1038/nrg2165.

23. Jurka J, Kapitonov VV, Pavlicek A, Klonowski P, Kohany O and Walichiewicz J. Repbase Update, a database of eukaryotic repetitive elements. Cytogenet Genome Res. 2005;110 1-4:462-7. doi:10.1159/000084979.

24. Tarailo-Graovac M and Chen N. Using RepeatMasker to identify repetitive elements in genomic sequences. Curr Protoc Bioinformatics. 2009;Chapter 4:Unit 4 10. doi:10.1002/0471250953.bi0410s25.

25. Burge C and Karlin S. Prediction of complete gene structures in human genomic DNA. J Mol Biol. 1997;268 1:78-94. doi:10.1006/jmbi.1997.0951.

26. Stanke M and Waack S. Gene prediction with a hidden Markov model and a new intron submodel. Bioinformatics. 2003;19 Suppl 2:ii215-25.

27. Majoros WH, Pertea M and Salzberg SL. TigrScan and GlimmerHMM: two open source ab initio eukaryotic gene-finders. Bioinformatics. 2004;20 16:2878-9. doi:10.1093/bioinformatics/bth315.

28. Blanco E, Parra G and Guigó R. Using geneid to identify genes. Current Protocols in Bioinformatics. 2007;18 1:4.3.1-4.3.28.

29. Korf I. Gene finding in novel genomes. BMC Bioinformatics. 2004;5:59. doi:10.1186/1471-2105-5-59.

30. Keilwagen J, Wenk M, Erickson JL, Schattat MH, Grau J and Hartung F. Using intron position conservation for homology-based gene prediction. Nucleic Acids Res. 2016;44 9:e89. doi:10.1093/nar/gkw092.

31. Bai CM, Rosani U, Xin LS, Li GY, Li C, Wang QC, et al. Dual transcriptomic analysis of Ostreid herpesvirus 1 infected *Scapharca broughtonii* with an emphasis on viral anti-apoptosis activities and host oxidative bursts. Fish Shellfish Immun. 2018;82:554-64.

doi:10.1016/j.fsi.2018.08.054.

32. Campbell MA, Haas BJ, Hamilton JP, Mount SM and Buell CR. Comprehensive analysis of alternative splicing in rice and comparative analyses with Arabidopsis. BMC Genomics. 2006;7:327. doi:10.1186/1471-2164-7-327.

33. Pertea M, Kim D, Pertea GM, Leek JT and Salzberg SL. Transcript-level expression analysis of RNA-seq experiments with HISAT, StringTie and Ballgown. Nat Protoc. 2016;11 9:1650-67. doi:10.1038/nprot.2016.095.

34. Tang S, Lomsadze A and Borodovsky M. Identification of protein coding regions in RNA transcripts. Nucleic Acids Res. 2015;43 12:e78. doi:10.1093/nar/gkv227.

35. Haas BJ, Salzberg SL, Zhu W, Pertea M, Allen JE, Orvis J, et al. Automated eukaryotic gene structure annotation using EVidenceModeler and the Program to Assemble Spliced Alignments. Genome Biol. 2008;9 1:R7. doi:10.1186/gb-2008-9-1-r7.

36. Birney E, Clamp M and Durbin R. GeneWise and Genomewise. Genome Res. 2004;14 5:988-95. doi:10.1101/gr.1865504.

37. Marchler-Bauer A, Lu S, Anderson JB, Chitsaz F, Derbyshire MK, DeWeese-Scott C, et al. CDD: a Conserved Domain Database for the functional annotation of proteins. Nucleic Acids Res. 2011;39 Database issue:D225-9. doi:10.1093/nar/gkq1189.

38. Boeckmann B, Bairoch A, Apweiler R, Blatter MC, Estreicher A, Gasteiger E, et al. The SWISS-PROT protein knowledgebase and its supplement TrEMBL in 2003. Nucleic Acids Res. 2003;31 1:365-70.

39. Tatusov RL, Natale DA, Garkavtsev IV, Tatusova TA, Shankavaram UT, Rao BS, et al. The COG database: new developments in phylogenetic classification of proteins from complete genomes. Nucleic Acids Res. 2001;29 1:22-8.

40. Kanehisa M and Goto S. KEGG: kyoto encyclopedia of genes and genomes. Nucleic Acids Res. 2000;28 1:27-30.

41. Altschul SF, Gish W, Miller W, Myers EW and Lipman DJ. Basic local alignment search tool. J Mol Biol. 1990;215 3:403-10. doi:10.1016/S0022-2836(05)80360-2.

42. El-Gebali S, Mistry J, Bateman A, Eddy SR, Luciani A, Potter SC, et al. The Pfam protein families database in 2019. Nucleic Acids Res. 2018; doi:10.1093/nar/gky995.

43. Eddy SR, Mitchison G and Durbin R. Maximum discrimination hidden Markov models of sequence consensus. J Comput Biol. 1995;2 1:9-23. doi:10.1089/cmb.1995.2.9.

44. Dimmer EC, Huntley RP, Alam-Faruque Y, Sawford T, O'Donovan C, Martin MJ, et al. The UniProt-GO Annotation database in 2011. Nucleic Acids Res. 2012;40 Database issue:D565-70. doi:10.1093/nar/gkr1048.

45. Conesa A, Gotz S, Garcia-Gomez JM, Terol J, Talon M and Robles M. Blast2GO: a universal tool for annotation, visualization and analysis in functional genomics research. Bioinformatics. 2005;21 18:3674-6. doi:10.1093/bioinformatics/bti610.

46. Griffiths-Jones S, Moxon S, Marshall M, Khanna A, Eddy SR and Bateman A. Rfam: annotating non-coding RNAs in complete genomes. Nucleic Acids Res. 2005;33 Database issue:D121-4. doi:10.1093/nar/gki081.

47. Griffiths-Jones S, Grocock RJ, van Dongen S, Bateman A and Enright AJ. miRBase: microRNA sequences, targets and gene nomenclature. Nucleic Acids Res. 2006;34 Database issue:D140-4. doi:10.1093/nar/gkj112.

48. Nawrocki EP and Eddy SR. Infernal 1.1: 100-fold faster RNA homology searches. Bioinformatics. 2013;29 22:2933-5. doi:10.1093/bioinformatics/btt509.
49. Lowe TM and Eddy SR. tRNAscan-SE: a program for improved detection of transfer RNA genes in genomic sequence. Nucleic Acids Res. 1997;25 5:955-64.

Figure legends

Figure 1: Hi-C interaction heat map for *Scapharca* (*Anadara*) *broughtonii*.

Figure 2: Gene ontology (GO) annotation of the predicted genes.
The horizontal axis indicates classes of the second level GO annotation. The vertical axis indicates the number and percentage of genes in each class.

Figure 3: Eukaryotic Orthologous Groups (KOG) classification of the predicted genes.
Results are summarized in 24 function classes according to their functions. The horizontal axis represents each class, and the vertical axis represents the frequency of the classes.

 Click here to access/download;Table;Table 1R.docx

Table 1. Summary of sequencing data generated for bloody clam genome assembly and annotation

Library type	Platform	Library size (bp)	Data size (Gb)	Application
Short reads	HiSeq X Ten	350	53.06	Genome survey, correction and evaluation
Long reads	PacBio SEQUEL	20,000	63.33	Genome assembly
	PacBio RS II	20,000	3.99	
	Nanopore Minion	20,000	8.47	
Hi-C	HiSeq X Ten	350	52.16	Chromosome construction

Table 2 Click here to access/download;Table;Table 2.docx

Table 2. Statics of the final genome assembly of *Scapharca* (*Anadara*) *broughtonii*

Types	Number	Length (bp)	N50 (bp)	N90 (bp)	Max (bp)	GC content	Gap (bp)
Scaffold	1026	884,566,040	44,995,656	25,444,477	55,667,740	33.70 %	65,100
Contig	1,667	884,500,940	1,797,717	305,905	7,852,409	33.70 %	0

 Click here to access/download;Table;Table 3.docx

Table 3. Statics of gene annotation to different databases

Annotation database	Annotated number	Percentage (%)
GO_Annotation	5,766	23.98%
KEGG_Annotation	9,174	38.15%
KOG_Annotation	13,626	56.67%
Pfam_Annotation	17,321	72.04%
Swissprot_Annotation	12,866	53.51%
TrEMBL_Annotation	21,887	91.03%
nr_Annotation	21,897	91.07%
nt_Annotation	12,786	53.18%
All_Annotated	22,308	92.78%

Figure 1

Click here to access/download;Figure;Figure1 Hi-C heatmap_for_final.jpeg

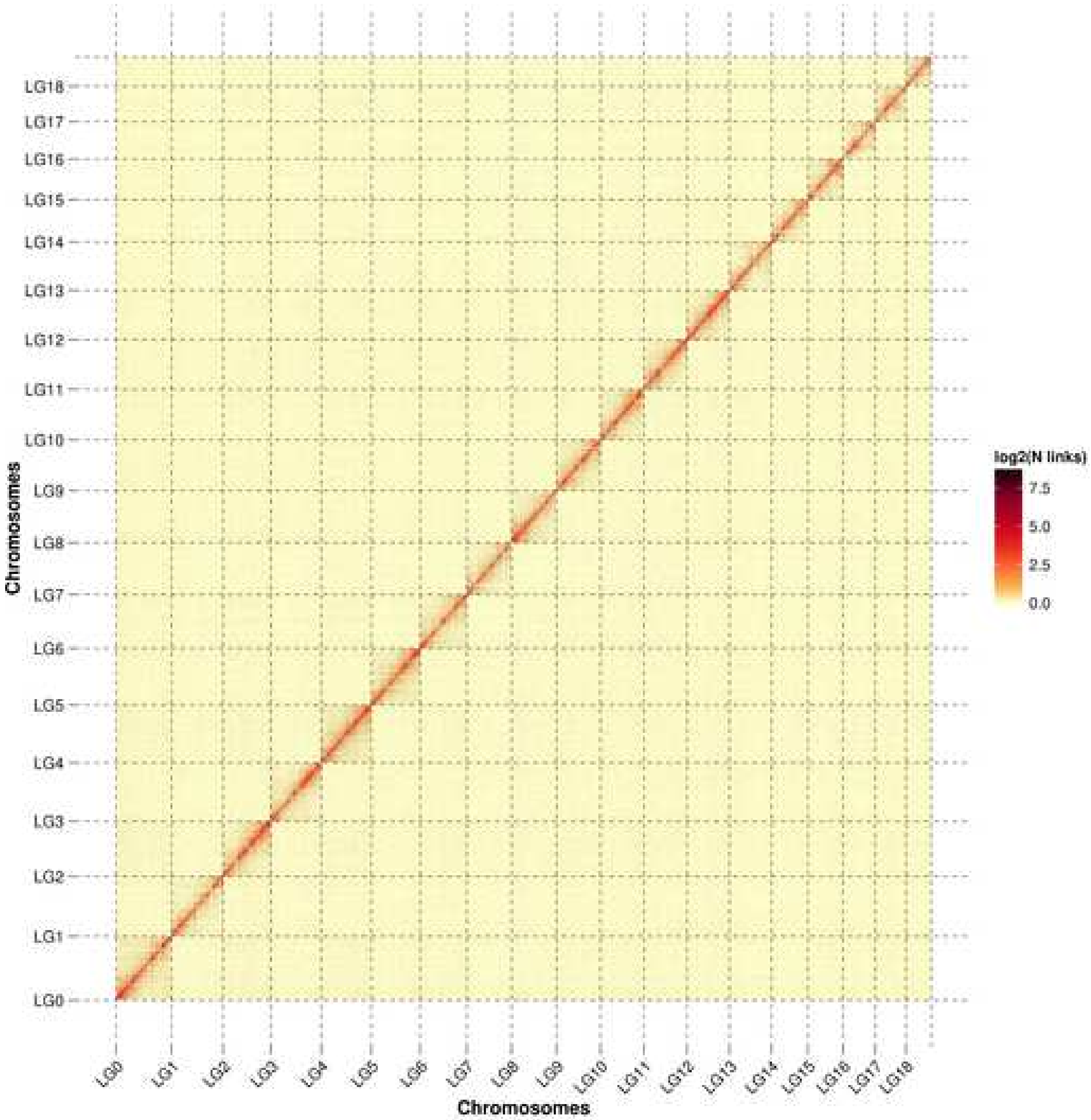

Figure 2

Click here to access/download;Figure;Figure2

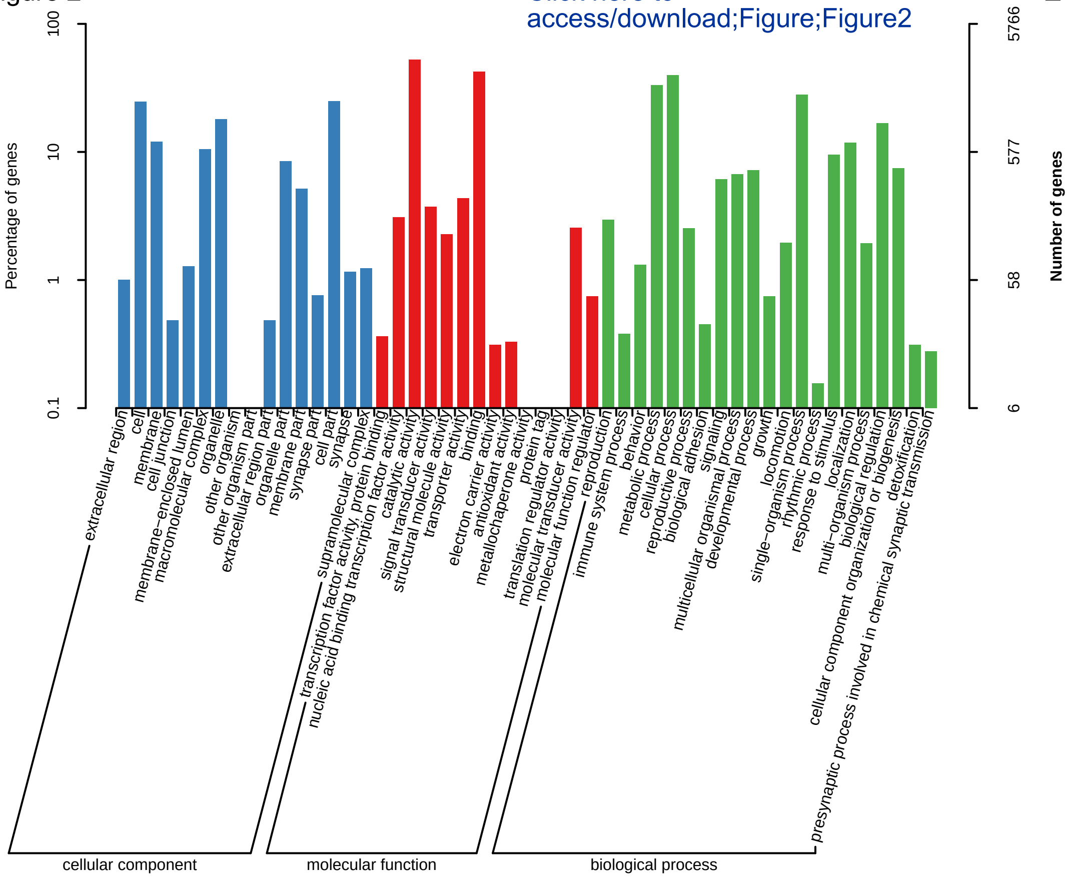

Figure 3

Click here to access/download;Figure;Figure3 EVM_GFF3.Kog.cluster.tif

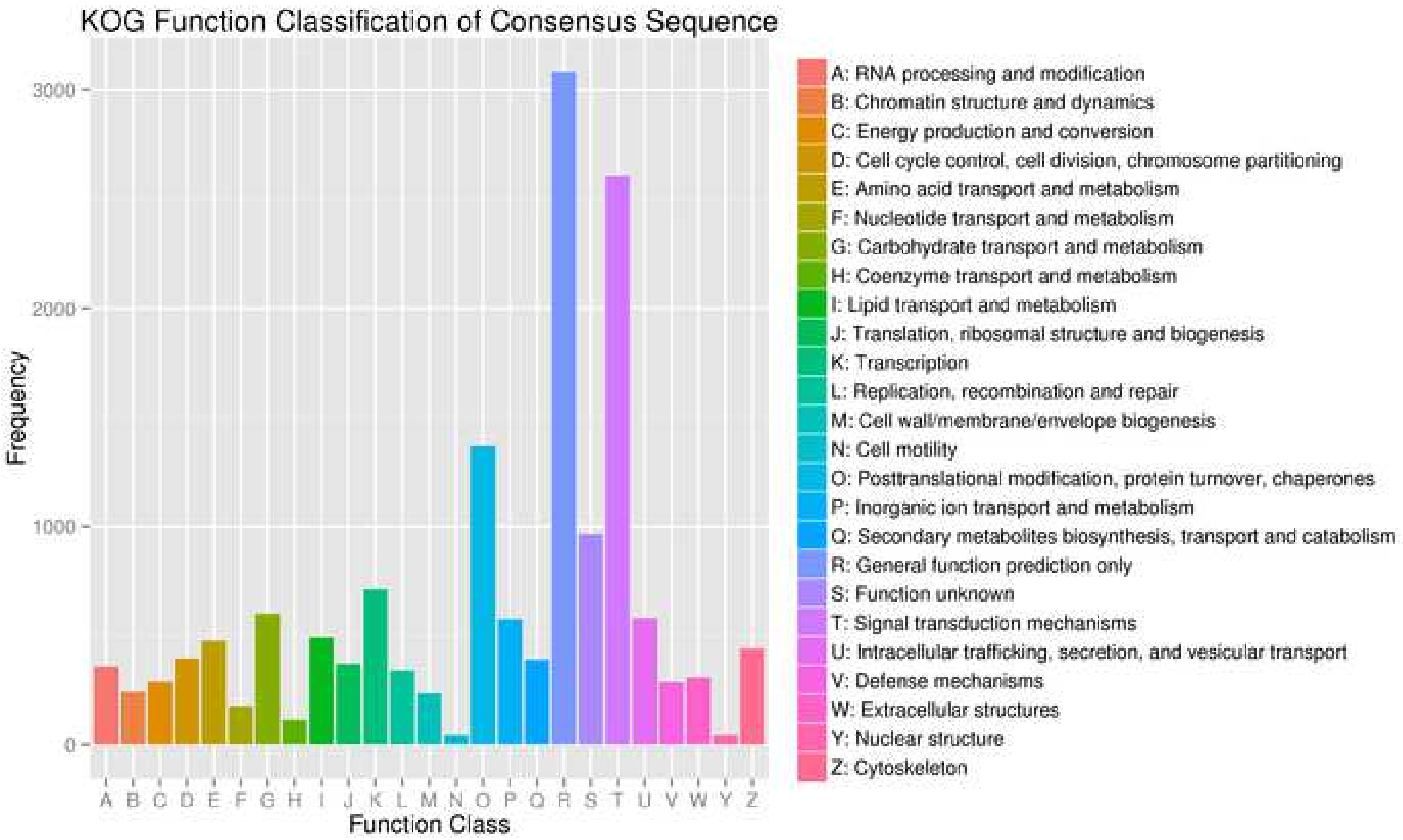

Click here to access/download
Supplementary Material
Supplementary material.docx

Click here to access/download
Supplementary Material
Supplementary Table11.xlsx

Cover letter Click here to access/download;Personal Cover;Cover letter.docx

Dr. Laurie Goodman

Editor in Chief

GigaScience

Feb 12, 2019

Dear Dr. Goodman

We are pleased to submit a manuscript entitled "Chromosomal-level assembly of the bloody clam, *Scapharca (Anadara) broughtonii*, using long sequence reads and Hi-C" for consideration for publication in *GigaScience*. We confirm that this manuscript has not been published elsewhere. This is the first *de novo* sequencing and assembly of genome sequence belonging to the Family Arcidae, Phylum Mollusca, which provides a rich resource for genomic studies.

We sequenced the bloody clam genome with the Pacbio and Nanopore platforms and generated a total of 75.79 Gb long-reads data representing approx. 86× coverage of the genome. *De novo* assembly of the long reads generated an 884.5 Mb genome with a contig N50 of 1.80 Mb and scaffold N50 of 45.00 Mb, respectively. Hi-C scaffolding of the genome resulted in 19 chromosomes containing 99.35% bases of the assembled genome. Genome annotation revealed that a considerable part of the genome (46.1%) is composed by repeated sequences. Gene prediction identified 24,045 protein-coding genes, and 84.7% of them were annotated in at least one database.

The raw data has been submitted to NCBI SRA database under the PRJNA521075, and a reviewer link to metadata was provided as: ftp://ftp-trace.ncbi.nlm.nih.gov/sra/review/SRP183816_20190206_170212_37d5c0b6b354bc3c790d2696b42756c9. The assembled and analysis results were also transferred to you under the FTP address: ftp://user95@parrot.genomics.cn,

which could be found with the following credentials, **username: user95 and password: WangCMClam**.

We recommended the following researchers as potential reviewers for the manuscript:

1. Marta Gomez Chiarri, E-mail: gomezchi@uri.edu, Website: https://web.uri.edu/favs/marta-gomez-chiarri/
2. Bassem Allam, E-mail: bassem.allam@stonybrook.edu, Website: https://you.stonybrook.edu/madl/people/faculty/bassem-allam/
3. Ximing Guo, E-mail: xguo@hsrl.rutgers.edu, Website: https://marine.rutgers.edu/main/ximing-guo
4. Rebeca Moreira, E-mail: rebecamoreira@iim.csic.es, ORCID: https://orcid.org/0000-0001-7797-7221

Thanks for your consideration of our manuscript. I look forward to hearing from you.

Sincerely,

Prof. Chong-Ming Wang

Yellow Sea Fisheries Research Institute (YSFRI)

E-mial: wangcm@ysfri.ac.cn
