# Supplementary material for: Chromosomal-level assembly of the blood clam, Scapharca (Anadara) broughtonii, using long sequence reads and Hi-C
Source: Gigascience. 2019 Jul 9;8(7):giz067. doi: 10.1093/gigascience/giz067 (PMC6615981; doi:10.1093/gigascience/giz067)
Supplement: giz067_GIGA-D-19-00008_Revision_1 [file giz067_giga-d-19-00008_revision_1.pdf]

GigaScience

Chromosomal-level assembly of the blood clam, Scapharca (Anadara) broughtonii, using long sequence reads and Hi-C

--Manuscript Draft--

**Manuscript Number:**	GIGA-D-19-00008R1
**Full Title:**	Chromosomal-level assembly of the blood clam, Scapharca (Anadara) broughtonii, using long sequence reads and Hi-C
**Article Type:**	Data Note
**Funding Information:**	China Agriculture Research System (CARS-49) — Prof. Chong-Ming Wang<br>National Key R&D Program of China (2018YFD0900304) — Dr. Biao Wu<br>National Natural Science Foundation of China (31602142) — Dr. Biao Wu<br>National Natural Science Foundation of China (31502208) — Dr. Chang-Ming Bai
**Abstract:**	Background: The blood clam, Scapharca (Anadara) broughtonii, is an economically and ecologically important marine bivalve of the family Arcidae. The efforts that have been made to study their population genetics, breeding, cultivation and stock enrichment were somewhat hindered by the lack of a reference genome. Here, we reported the complete genome sequence of S. broughtonii, a first reference genome of the family Arcidae.<br>Funding: A total of 75.79 Gb clean data was generated with the PacBio and Oxford Nanopore platforms, which represented approximately 86× coverage of the S. broughtonii genome. De novo assembly of these long reads resulted in an 884.5 Mb genome, with a contig N50 of 1.80 Mb and scaffold N50 of 45.00 Mb, respectively. Genome Hi-C scaffolding resulted in 19 chromosomes containing 99.35% of bases in the assembled genome. Genome annotation revealed that a considerable part of the genome (46.1%) is composed by repeated sequences, while 24,045 protein-coding genes were predicted and 84.7% of them were annotated.<br>Conclusion: We report here the chromosomal-level assembly of the S. broughtonii genome based on long read sequencing and Hi-C scaffolding. The genomic data could be served as reference genome for family Arcidae and will provide a valuable resource for the scientific community and aquaculture sector.
**Corresponding Author:**	Chong-Ming Wang<br>YSFRI<br>Qingdao, Shandong CHINA
**Corresponding Author Secondary Information:**	
**Corresponding Author's Institution:**	YSFRI
**Corresponding Author's Secondary Institution:**	
**First Author:**	Chang-Ming Bai, Ph.D.
**First Author Secondary Information:**	
**Order of Authors:**	Chang-Ming Bai, Ph.D.<br>Lu-Sheng Xin<br>Umberto Rosani<br>Biao Wu<br>Qing-Chen Wang<br>Xiao-Ke Duan

	Zhi-Hong Liu
	Chong-Ming Wang
**Order of Authors Secondary Information:**	
**Response to Reviewers:**	Dear GigaScience Editor,<br>We thank you and the two reviewers for the revision of our manuscript. We have read your requests/suggestions and those performed by the reviewers and each of these points have been revised carefully. We have added a figure (Figure 1) to show the shape and color of blood clam's shell and visceral mass. We have prepared a new supplementary table (the new Supplementary table 1) to present the key protocols, that were also uploaded to protocols.io as suggested by you. except that of Hi-C library preparation, We have provided more details of the key steps of Hi-C library construction at Lines 111-120. However, we do not include the very detailed Hi-C library protocol in Supplementary table 1, since it is a business secret of the BioMarker company, and they are still reluctant to provide us these parameters.<br>Here, we provide a point-by-point response to each reviewer's comments.<br><br>Sincerely,<br>Chong-Ming Wang<br>Yellow Sea Fisheries Research Institute (YSFRI)<br>E-mial: wangcm@ysfri.ac.cn<br><br>Reviewer #1<br>General comments:<br>Functional annotation was fairly extensive through the BLASTing of protein sequences to multiple databases. A statement should be added about the nr annotation as the nr database is not manually curated and is known to have errors that can be propogated. "Functional annotations that are found only in the nr database should not be used to annotate new genomes."<br>Following reviewer's comments, we have found and removed from the annotation table 41 genes annotated only in the Nr database. Now, we presented a final set of 22, 267 annotated genes.<br>The supplemental table with the blast annotations only contain the functional annotation without information about the blast score, length of alignment etc. It would be of great value to this data note if this information was added.<br>Following reviewer's comments, we have added blast score, length of alignment et al. for blast annotations to Nr and Nt databases. The detailed information regarding the functional annotations to each database has been submitted to the GigaScience database (ftp://user95@parrot.genomics.cn) and it can be accessed by the reviewers using our credentials (user: user95 and password: WangCMClam). We prefer to not include all these details to the annotation supplemental table, since it will become difficult to read because of its large size.<br>Specific comments:<br>1) If available please state the number of places where Hi-C broke contigs in the assembly.<br>There are 343 broke points during the Hi-C scaffolding process, detailed information has been uploaded to GigaScience database (ftp://user95@parrot.genomics.cn). We have stated this point at Line 136 in the main text.<br>2) For all programs used please state the verson and all parameters required to replicate your analysis<br>We have provided the versions and parameters of all programs used in the manuscript at protocols.io and in the new Supplementary Table 1.<br>3) For all databases used (Kegg, nr KOG etc) please state the version or date of download used in annotation.<br>We have provided the version or date of download of all databases used in the manuscript at protocols.io and in the new Supplementary Table 1.<br>4) For the Blast analysis please specify if you used max-target-seq in your BLAST analysis and if you took the Best Blast Hit. How did you decide which Annotation to use?<br>Yes, we used the max-target-seq in our BLAST analysis with the parameter: -max_target_seqs 100 (we have specified this point at protocols.io and in the new Supplementary Table 1.). For the final annotation we have selected the annotation with the highest score

5) Please specify which Illumina reads were used during Pilon polishing.
The illumina reads for genome survey was used during Pilon polishing. We have stated this point at Line 98.
6) Would prefer that the authors include the blast result for each annotation provided in the supplemental table 11.
Please see the general comments.
Line 51: The word knew should be know. "Compared to oysters and scallops, we still know very little ..."
The section containing “knew” have been revised as a whole.

Reviewer #2
Major points:
The English of the manuscript is poor. In most places where there are issues, it is just awkward but in some places the meaning is not clear.
We have invited a native speaker to kindly revise the language of the manuscript thoroughly. He fixed several language pitfalls and now we hope that the overall language quality is acceptable.
Was the DNA / material used all from the same individual?
We used haemocytes collected from several specimens for DNA extraction, to obtain enough DNA for the different libraries we constructed. We have specified this point in the main text at line 60.
How were the reads filtered (line 91)?
We used a custom perl script to filter the reads shorter than 500 bp. We have stated this point at Lines 93-94.
How many cycles of Pilon were used?
We used three cysles, we have stated this at lines 97-98.
What were the BUSCO results of the merged assembly before removal of redundancy with Numer? Were other tools such as Redundans explored for redundancy reduction?
We reduced the redundancy under the premise of keeping the integrity of the data. The evaluations of the different intermediate datasets were not included, while we displayed the best result. We did not use Redundans or other tools for redundancy reduction, since we were satisfied with the performance of Numer.
Methods used for Hi-C library preparation are inadequate.
We have provided more detailed information about the Hi-C library preparation at lines 111-120. We were not allowed to include some parameters because the protocol is a business secret of the BioMarker company, and they are reluctant to provide us these data.
The procedure described on lines 124-125 is not well explained. Why was this performed?
We have revised this section to provide more details and reasonability about Hi-C assembly at Lines 133-136.
Line 157: "the results of the three approaches" - unclear which three steps are referred to.
This refers to ‘ab initio prediction, homology-based prediction, and transcriptome-based prediction’, We have revised this point at Line 168 to make it more clear.
Lines 160-161: The procedure to detect pseudogenes is not adequately described.
We have revise this section at Lines 171-177.
Availability of Data and Materials - what about the predicted transcripts and protein sequences?
These information has been uploaded to GigaScience database (ftp://user95@parrot.genomics.cn) and can be accessed by the reviewers using our credentials (see answer to Rew1), whereas they will immediately released after manuscript acceptance.

Minor points:
Line 38: To my knowledge, "ark shell" is a common name used for the entire family Arcidae, not just this species.
We agree with you that "ark shell" is a common name used for the family Arcidae. The Scapharca (Anadara) broughtonii is always called ‘blood clam’ or ‘bloody clam’ in publications and Asia countries where the species is mainly distributed. So, we have revised this point indicating that ‘blood clam’ is a species of ‘ark shell’ (Lines 41-42).
Line 40: Correct "lived" to "lives"
We have replaced "lived" with "lives" at line 43.

	Line 43: Correct "mollusk" to "molluscs"<br>We have replaced "mollusk" with "molluscs" at line 46.<br>Line 61: Correct "libraries" to "library"<br>We have replaced " libraries " with "library" at line 66.
**Additional Information:**	
**Question**	**Response**
Are you submitting this manuscript to a special series or article collection?	No
**Experimental design and statistics**<br><br>Full details of the experimental design and statistical methods used should be given in the Methods section, as detailed in our Minimum Standards Reporting Checklist. Information essential to interpreting the data presented should be made available in the figure legends.<br><br>Have you included all the information requested in your manuscript?	Yes
**Resources**<br><br>A description of all resources used, including antibodies, cell lines, animals and software tools, with enough information to allow them to be uniquely identified, should be included in the Methods section. Authors are strongly encouraged to cite Research Resource Identifiers (RRIDs) for antibodies, model organisms and tools, where possible.<br><br>Have you included the information requested as detailed in our Minimum Standards Reporting Checklist?	Yes
**Availability of data and materials**<br><br>All datasets and code on which the conclusions of the paper rely must be either included in your submission or deposited in publicly available repositories (where available and ethically appropriate), referencing such data using	Yes

Powered by Editorial Manager® and ProduXion Manager® from Aries Systems Corporation

a unique identifier in the references and in the “Availability of Data and Materials” section of your manuscript.<br><br>Have you have met the above requirement as detailed in our Minimum Standards Reporting Checklist?	

Manuscript

Click here to access/download;Manuscript;008-ArkshellGenome_23Apr-HL.docx

Click here to view linked References

Chromosomal-level assembly of the blood clam, *Scapharca* (*Anadara*) *broughtonii*, using long sequence reads and Hi-C

Chang-Ming Bai[a]†, Lu-Sheng Xin[a]†, Umberto Rosani[b,c], Biao Wu[a], Qing-Chen Wang[a], Xiao-Ke Duan[d], Zhi-Hong Liu[a], Chong-Ming Wang[a]*

[a] Key Laboratory of Maricultural Organism Disease Control, Ministry of Agriculture; Laboratory for Marine Fisheries Science and Food Production Processes, Qingdao National Laboratory for Marine Science and Technology; Qingdao Key Laboratory of Mariculture Epidemiology and Biosecurity; Yellow Sea Fisheries Research Institute, Chinese Academy of Fishery Sciences, Qingdao 266071, China

[b] Department of Biology, University of Padua, Padua 35121, Italy

[c] Alfred Wegener Institute – Helmholtz Centre for Polar and Marine Research, Wadden Sea Station Sylt, Germany

[d] Biomarker Technologies Corporation, Beijing 101200, China

† These authors contributed equally to this work.

* Correspondence to: Chong-Ming Wang, E-mail address: wangcm@ysfri.ac.cn.

Chang-Ming Bai, ORCID: 0000-0001-5476-2455;

Lu-Sheng Xin, ORCID: 000-0002-4685-1076;

Umberto Rosani, ORCID: 0000-0003-0685-1618;

Biao Wu, ORCID: 0000-0002-6522-8140;

Chong-Ming Wang, ORCID: 0000-0002-5517-9453.

Abstract

Background: The blood clam, *Scapharca* (*Anadara*) *broughtonii,* is an economically and ecologically important marine bivalve of the family Arcidae. Efforts that have been made to study their population genetics, breeding, cultivation and stock enrichment have been somewhat hindered by the lack of a reference genome. Here, we reported the complete genome sequence of *S. broughtonii,* a first reference genome of the family Arcidae.

Funding: A total of 75.79 Gb clean data was generated with the PacBio and Oxford Nanopore platforms, which represented approximately 86× coverage of the *S. broughtonii* genome. *De novo* assembly of these long reads resulted in an 884.5 Mb genome, with a contig N50 of 1.80 Mb and scaffold N50 of 45.00 Mb, respectively. Genome Hi-C scaffolding resulted in 19 chromosomes containing 99.35% of bases in the assembled genome. Genome annotation revealed that a considerable part of the genome (46.1%) is composed by repeated sequences, while 24,045 protein-coding genes were predicted and 84.7% of them were annotated.

Conclusion: We report here the chromosomal-level assembly of the *S. broughtonii* genome based on long read sequencing and Hi-C scaffolding. The genomic data can serve as a reference for the family Arcidae and will provide a valuable resource for the scientific community and aquaculture sector.

Keywords: ark shell; PacBio; Hi-C; genomic; chromosomal assembly.

Background information

The blood clam, *Scapharca* (*Anadara*) *broughtonii* (Schrenck, 1867; NCBI: txid148819; marinespecies.org:taxname:591364), is a species of ark shell of the family Arcidae, class Pteriomorphia, phylum Mollusca. Although most of the approximately 200 species of the family Arcidae are distributed in tropical areas [1], *S. broughtonii* lives in temperate areas along the coasts of Northern China, Japan, Korea and the Russian Far East [1, 2]. The name "blood clam" originated from the red color of their visceral mass, due to the presence of hemoglobin in both tissues and hemolymph [1, 2], a rare trait in molluscs, but a hallmark of Arcidae species [3]. *S. broughtonii* specimens are characterized by thick and harder calcareous shells, covered by a hairy brown colored periostracum colored (Figure 1) [2]. Adult blood clams can reach a shell length of 100 mm [4] and they are harvested as a source of sashimi, which as bring to the depletion of the wild resources in the last century. Many efforts have been made to recover the wild population stocks of *S. broughtonii* in China, Japan and Korea, including intensive farming. Such aquaculture practices have revealed the susceptibility of *S. broughtonii* to many pathogenic bacteria and viruses, including a variant of the *Ostreid herpesvirus 1* [1, 5-7]. Compared to other aquaculture-important bivalve species, like oysters, mussels and scallops, the genomic and transcriptomic resources of Arcidae species are still limited. Therefore, the understanding of their basic biological processes as well as of more complex host-pathogen interactions is somewhat hampered. Here, we sequenced the complete genome of *S. broughtonii* at the chromosomal level and we offer it as a valuable resource to develop both scientific research and aquaculture industry related to Arcidae species.

Sample collection and sequencing

Adult *S. broughtonii* specimens were sampled from populations near Jimo, Shandong Province, China. To overcome the excessive polysaccharide content of *S. broughtonii* tissues, high-quality genomic DNA was extracted from haemocytes, using DNeasy® Blood & Tissue Kit (Qiagen, p/n 69504) with a few protocol modifications to remove polysaccharides (the detailed protocol is reported at protocols.io [8] and Supplementary Table S1). The DNA quantity and quality were measured with Qubit 3.0 (Thermo Fisher Scientific Inc., Carlsbad, CA, USA) and agarose gel electrophoresis, respectively. High-quality DNA was used for library preparation and high-throughput sequencing using PacBio, Nanopore and Illumina platforms (Table 1, BioMarker Technology Co. Ltd., Beijing,

China).

PacBio sequencing was carried out with the SMRT Bell™ library using a DNA Template Prep Kit 1.0 (Pacific Biosciences, Menlo Park, CA, USA, p/n 100-259-100). All the detailed library preparation protocols are available on protocols.io [9]. Briefly, the genomic DNA (10 μg) was mechanically sheared using a Covaris g-Tube (Kbiosciences p/n 520079) to get DNA fragments of approx. 20 Kb in size. The sheared DNA was DNA-damage and end-repaired using polishing enzymes. Then a blunt-end ligation reaction followed by exonuclease treatment was conducted to generate the SMRT Bell™ template. Finally, large fragments (>10 Kb) were enriched with Blue Pippin device (Sage Science, Inc., Beverly, MA, USA) for sequencing. A total of 15 SMRT cells were processed, 7 with Sequel and 8 with RS II instruments (Pacific Biosciences), to generate a total of 67.32 Gb PacBio data.

For Oxford Nanopore sequencing, approx. 5 μg of genomic DNA was sheared and size-selected (~20 kb) with the same procedure described above. The selected fragments were processed using the Ligation Sequencing 1D Kit (Oxford Nanopore, Oxford, UK, p/n SQK-LSK109) according to the manufacturer's instructions and sequenced using the MinION portable DNA sequencer with the 48 hours run script (Oxford Nanopore), to generate a total of 8.47 Gb data. For Illumina sequencing, paired-end (PE) library with an insert size of 350 bp was constructed according to the manufacturer's protocol, and sequenced with an Illumina HiSeq X Ten platform (Illumina Inc. San Diego, CA, USA) with paired-end 150 (PE150) read layout. A total of 53.06 Gb Illumina data was generated and used for genome survey, correction and evaluation (Supplementary Table S2). All high-throughput sequencing data have been deposited at the NCBI SRA database under accession ID SAMN10879241.

Initial genome assembly and evaluation

The Sequel and RS II raw files (bam and H5 formats) were converted into subreads in fasta format with the standard PacBio SMRT software package, for a total of 63,330,577,481 and 3,990,849,516 base pairs (bp), respectively. Subreads shorter than 500 bp in size were filtered out, to obtain a clean dataset of 4,761,097 PacBio reads for a total of 67,260,156,459 bp, with a read N50 of 21,932 and a mean read length of 14,127 bp (Supplementary Table S3). The Nanopore reads were base-called from the raw FAST5 files using Guppy implanted in MinKNOW (Oxford Nanopore), applying a minimum length cutoff of 500 bp, for a total of 8,468,912,896 bp, with a read N50 of 20,804 and a read mean length of 15,143 bp (Supplementary Table S4). Hybrid assembly of the clean reads were carried out

using Canu v1.5 (Canu, RRID:SCR_015880) [10] and WTDBG v1.1 [11] tools. The two assemblies were joined using Quickmerge v0.2.2 [12] and the redundancy was removed with Numer v4.0.0 [13]. Finally, the genome assembly was corrected for 3 cycles with the Illumina reads prepared specifically for genome survey using Pilon v1.22 (Pilon, RRID: SCR 014731) with default settings [14]. This initial genome assembly was 884,500,940 bp in length with a contig N50 of 2,388,811 bp (Supplementary Table S5). The detailed parameters of each tool used for genome assembly are available at protocols.io [15].

We evaluated the quality of the initial assembly by mapping the 360,937,442 Illumina reads for genome survey to the assembly using SAMTools v0.1.18 (SAMTOOLS, RRID:SCR_002105) [16], and by searching the 303 eukaryotic and 978 metazoan conserved genes in the assembly using BUSCO v2.0 (BUSCO, RRID:SCR 015008) [17]. As a result, 97.45 % of the Illumina reads were successfully mapped to the assembled genome. The BUSCO analysis found 273 and 897 conserved genes belonging to eukaryote and metazoan datasets, accounting for 90.10 % and 91.72 % of the totals, respectively (Supplementary Table S6). These results indicated the considerable quality of this initial genome assembly of *S. broughtonii*.

Hi-C analysis and chromosome assembly

Fresh adductor muscle collected from a single *S. broughtonii* specimen of the same population was firstly fixed using formaldehyde with a final concentration of 1%. The fixed tissue was then homogenized with tissue lysis, digested with the restriction enzyme (*Hind* III), *in situ* labeled with a biotinylated residue and end-repaired. Finally, the DNA was extracted and used for Hi-C library preparation using the Nextera Mate Pair Sample Preparation Kit (Illumina, p/n FC-132-1001). Briefly speaking, 5-6 μg DNA was firstly sheared, end-repaired, selected for fragments with a length of 300-700 bp, and captured the biotin-containing fragments. Then the basic standard steps of dA-tailing, adapter ligation, PCR amplification and purification were carried out. Finally, the quality of purified library was evaluated with Qubit 3.0 (Thermo Fisher Scientific Inc.), quantitative PCR (Q-PCR) and Caliper LabChip GX Analyzer (Waltham, MA, USA). The qualified library was sequenced using an Illumina HiSeq X Ten platform with 150 PE layout. A total of 174,148,156 read pairs (52.16 Gb) with a Q30 of 93.16% were generated and used for the subsequent Hi-C analysis (NCBI SRA accession number: SAMN10879242).

To get the unique mapped read pairs, the 174 million read pairs were first truncated at the putative Hi-C junctions and then aligned to the *S. broughtonii* genome assembly using the BWA aligner v0.7.10-r789 (BWA, RRID:SCR_010910) [18]. A total of 206 million reads (59.23%) mapped to the assembled genome, of which 51 million read pairs (29.33%) showed unique mapped read pairs (Supplementary Table S7). Only the uniquely aligned pairs with a mapping quality higher than 20 were further considered, while the invalid interaction pairs due to self-circle ligation, dangling ends, re-ligation and the other dumped types were filtered out with HiC-Pro v2.10.0 [19]. A total of 17 million valid interaction pairs, accounting for 33.66% of the unique mapped read pairs (Supplementary Table S8) were used for the Hi-C analysis. Detailed Hi-C assembly parameters are available at protocols.io [20].

To correct mis-assemblies occurred in the initial assembly, the contigs were broken into 300 bp fragments and then assembled based on Hi-C data using Lachesis v2e27abb [21]. The genomic regions characterized by the sudden drop of physical coverage were defined as mis-assemblies and contigs were broken at that point [22]. As a result, we identified 343 break points in 156 contigs, and 1,645 corrected contigs with a N50 of 1.81 Mb and a length of 884.50 Mb. Then the corrected contigs were reassembled using Lachesis. Finally, 1,384 contigs (82.53%) were successfully clustered into 19 groups (Figure 2), which was consistent with previous karyotype analyses of *S. broughtonii* [23]. The 1,384 clustered contigs correspond to a length of 878.79 Mb (99.35 % of the length of the corrected contigs). Among the 1,384 clustered contigs, 670 contigs (819.17 Mb) were anchored with defined order and orientation, accounting for 48.41% and 93.22% of the reassembled contigs by contig number and length, respectively (Supplementary Table S9). The final chromosomal-level *S. broughtonii* genome assembly, which represented the first reference genome of Family Arcidae, has a contig N50 of 1.80 Mb and scaffold N50 of 45.00 Mb (Table 2).

Genome annotation

We used LTR FINDER v1.05 (LTR_Finder, RRID:SCR_015247) [24], RepeatScout v1.0.5 (RepeatScout, RRID:SCR 014653) [25] and PILER-DF v2.4 [26] to construct a library of repetitive sequences based on the *S. broughtonii* genome. We classified these repeats using PASTEClassifier v1.0 [27] and we merged them with the Repbase database [28]. Finally, RepeatMasker v4.0.5 (RepeatMasker, RRID:SCR 012954) [29] was used to identify and mask the genomic repeated sequences for a total length of 407.8 Mb,

representing 46.1% of the total genome length. The statistics of amount, length and percentage of each repeat type could be found in Supplementary Table S10. Additional methodological information about genome annotation is available at protocols.io [15].

Protein-coding genes were predicted using the following approaches: *ab initio* prediction, homology-based prediction, and transcriptome-based prediction. For *ab initio* prediction, Genscan v3.1 (Genscan, RRID:SCR 012902) [30], Augustus v3.1 (Augustus, RRID:SCR 008417) [31], GlimmerHMM v1.2 (GlimmerHMM, RRID:SCR 002654) [32], GeneID v1.4 [33] and SNAP v2006-07-28 (SNAP, RRID:SCR 002127) [34] were used. For homology-based prediction, protein sequences of three closely related mollusc species (*Crassostrea gigas, Mizuhopecten yessoensis* and *Mytilus galloprovincialis*) and *Danio rerio* were downloaded from NCBI and aligned against the assembled genome with GeMoMa v1.3.1 [35]. For the transcriptome-based prediction, transcriptomic data obtained from a previous study (NCBI SRA accession ID: PRJNA450478) [36] were used as input data. In the previous study [36], RNA-seq data had been *de novo* assembled with Trinity v.r20140413p1 and the gene predictions were carried out with PASA v2.0.2 (PASA, RRID:SCR_014656) [37]. We also performed reference-based assembly of the RNA-seq data with Hisat v2.0.4 (HISAT2, RRID:SCR_015530) and Stringtie v1.2.3 [38], then we predicted the genes using TransDecoder v2.0 (http://transdecoder.github.io) and GeneMark v5.1 (GeneMark, RRID:SCR_011930) [39]. All the gene predictions were integrated using EVM v1.1.1 (EVM, RRID:SCR 014659) [40], and further modified with PASA v2.0.2, to obtain a final dataset of 24,045 predicted genes with an average length of 12,549 bp (Supplementary Table S11).

Pseudogenes emerge from coding genes that have become non-functional due to accumulation of mutations [41, 42]. A sequence that is homologous to a normal protein-coding gene but not annotated as protein-coding genes is likely to be a pseudogene. Therefore, based on homology to known protein-coding genes, putative pseudogenes were firstly searched in the intergenic regions of the *S. broughtonii* genome using genBlastA v1.0.4 [43]. Then GeneWise v2.4.1 (GeneWise, RRID:SCR 015054) [44] was adopted to search the premature stop codons or frameshift mutations in those sequences and to finally identify a total of 1,658 pseudogenes, with an average length of 3,151 bp.

The predicted genes were annotated by aligning them to the NCBI non-redundant protein (nr) [45], non-redundant nucleotide (nt) [45], Swissprot (Swissprot, RRID:SCR 002380) [46], TrEMBL (TrEMBL,

RRID:SCR 002380) [46], KOG [47] and KEGG (KEGG, RRID:SCR 001120) [48] databases using BLAST v2.2.31 [49] with a maximal e-value of $1e^{-5;}$ by aligning to the Pfam database (Pfam, RRID:SCR 004726) [50] using HMMer V3.0 [51]. Gene Ontology (GO) terms (Gene Ontology, RRID:SCR_002811) [52] were assigned to the genes using the BLAST2GO v2.5 pipeline (Blast2GO, RRID:SCR_005828) [53]. As a result, a total of 22, 267 genes were annotated in at least one database (Table 3, Supplementary Table S12). Among the 21,897 genes annotated in the nr database, 11,772 genes (53.7%) showed homology with *C. gigas* hits (Supplementary Figure S1). A total of 5,766 and 13,626 genes were annotated in GO and KOG databases, respectively, and the functional classification of these genes were presented in Figure 3 and 4, while the complete gene annotation table is reported in Supplementary Table 12.

Finally, we predicted the non-coding RNAs based on Rfam v12.1 (Rfam, RRID:SCR_007891) [54] and miRBase v21.0 (miRBase, RRID:SCR_003152) [55] databases. Putative miRNAs and rRNAs were predicted using Infernal v1.1 [56], tRNAs were predicted with tRNAscan-SE v1.3.1 (tRNAscan-SE, RRID:SCR_010835) [57]. A total of 27 miRNAs, 204 rRNAs, and 1,561 tRNAs were detected, corresponding to 15, 4, and 25 families, respectively.

Additional files

Supplementary Table S1. Key protocols for chromosome-level genome assembly of *Scapharca* (*Anadara*) *broughtonii*.

Supplementary Table S2. Summary of the Illumina sequencing reads used for genome survey, correction and evaluation.

Supplementary Table S3. Statics of the length distribution of Pacbio Subreads.

Supplementary Table S4. Statics of the length distribution of Oxford Nanopore reads.

Supplementary Table S5. Statics of the initial genome assembly of *Scapharca* (*Anadara*) *broughtonii.*

Supplementary Table S6. Summary of BUSCO analysis results.

Supplementary Table S7. Statistics of the mapping results of Hi-C reads.

Supplementary Table S8. Statistics of different types of the Hi-C reads.

Supplementary Table S9. Summary of the Hi-C assembly.

Supplementary Table S10. Statistics of the repeated sequences.

Supplementary Table S11. Summary of the gene prediction results.

Supplementary Figure S1. Species distribution of BLAST hits of the predicted genes in the NR database.

Supplementary Table S12. Integrated lists of gene annotation for the assembled *Scapharca* (*Anadara*) *broughtonii* genome.

Availability of Data and Materials

The DNA sequencing data and genome assembly have been deposited at the NCBI SRA database under the BioProject accession number PRJNA521075. Supporting data are also available via the *GigaScience* database GigaDB [58], and supporting protocols are archived in protocols.io [9].

Abbreviations

BLAST: Basic Local Alignment Search Tool; bp: base pair; BUSCO: Benchmarking Universal Single-Copy Orthologs; Gb: gigabase; GO: Gene Ontology; Hi-C: high-throughput chromosome conformation capture; KEGG: Kyoto Encyclopedia of Genes and Genomes; KOG: eukaryotic orthologous groups of proteins; Mb: megabase; NCBI: National Center for Biotechnology Information; PacBio: Pacific Biosciences; RNA-seq: RNA sequencing; SMRT: single-molecule real-time.

Competing interests

The authors declare that they have no competing interests.

Funding

This work was financially supported by National Key R&D Program of China (2018YFD0900304), China Agriculture Research System, grant number CARS-49, National Natural Science Foundation of China (31602142 and 31502208).

Author Contributions: C.W., C.B. and Q.W. conceived the project; C.W., C.B. and Q.W. collected the samples; C.B., L.X. and Q.W. extracted the genomic DNA and performed genome sequencing; C.B.,

L.X. X.D. and U.R. analyzed the data; U.R., B.W. and Z.L. participated in discussions and provided valuable advice; C.B., L.X., U.R., B.W. and Z.L. wrote and revised the manuscript.

Acknowledgements

We are grateful to Neal Scheraga for the language revision.

Reference:

1. An HY and Park JY. Ten new highly polymorphic microsatellite loci in the blood clam Scapharca broughtonii. Mol Ecol Notes. 2005;5 4:896-8. doi:DOI 10.1111/j.1471-8286.2005.01104.x.
2. Nishida K, Ishimura T, Suzuki A and Sasaki T. Seasonal changes in the shell microstructure of the bloody clam, *Scapharca broughtonii* (Mollusca: Bivalvia: Arcidae). Palaeogeogr Palaeocl. 2012;363:99-108. doi:10.1016/j.palaeo.2012.08.017.
3. Boyd SE. Order Arcoida. In: Beesley PL, Ross GJB and Wells A, editors. Mollusca: The Southern Synthesis Fauna of Australia, vol 5. Melbourne: CSIRO Publishing; 1998. p. 253–61.
4. Sugiura D, Katayama S, Sasa S and Sasaki K. Age And Growth Of the Ark Shell *Scapharca Broughtonii* (Bivalvia, Arcidae) In Japanese Waters. J Shellfish Res. 2014;33 1:315-24. doi:10.2983/035.033.0130.
5. Tang Q, Qiu X, Wang J, Guo X and Yang A. Resource enhancement of arkshell (*Scapharca (Anadara) broughtonii*) in Shandong offshore waters. Chinese Journal of Applied Ecology. 1994;5 4:396-402.
6. Bai C, Gao W, Wang C, Yu T, Zhang T, Qiu Z, et al. Identification and characterization of ostreid herpesvirus 1 associated with massive mortalities of *Scapharca broughtonii* broodstocks in China. Dis Aquat Organ. 2016;118 1:65-75. doi:10.3354/dao02958.
7. Zhao Q, Wu B, Liu Z, Sun X, Zhou L, Yang A, et al. Molecular cloning, expression and biochemical characterization of hemoglobin gene from ark shell *Scapharca broughtonii*. Fish Shellfish Immunol. 2018;78:60-8. doi:10.1016/j.fsi.2018.03.038.
8. Chang-Ming Bai, Lu-Sheng Xin, Umberto Rosani, Biao Wu, Qing-Chen Wang, Xiao-Ke Duan, et al. Extration of *Scapharca broughtonii* genomic DNA. protocols.io. 2019; https://dx.doi.org/10.17504/protocols.io.zhaf32e.
9. Chang-Ming Bai, Lu-Sheng Xin, Umberto Rosani, Biao Wu, Qing-Chen Wang, Xiao-Ke Duan, et al. Key protocols for chromosome-level genome assembly of the *Scapharca (Anadara) broughtonii.* protocols.io. 2019; https://dx.doi.org/10.17504/protocols.io.zimf4c6.
10. Koren S, Walenz BP, Berlin K, Miller JR, Bergman NH and Phillippy AM. Canu: scalable and accurate long-read assembly via adaptive k-mer weighting and repeat separation. Genome Res. 2017;27 5:722-36. doi:10.1101/gr.215087.116.
11. Jayakumar V and Sakakibara Y. Comprehensive evaluation of non-hybrid genome assembly tools for third-generation PacBio long-read sequence data. Brief Bioinform. 2017; doi:10.1093/bib/bbx147.
12. Chakraborty M, Baldwin-Brown JG, Long AD and Emerson JJ. Contiguous and accurate de novo assembly of metazoan genomes with modest long read coverage. Nucleic Acids Res. 2016;44 19:e147. doi:10.1093/nar/gkw654.
13. Kurtz S, Phillippy A, Delcher AL, Smoot M, Shumway M, Antonescu C, et al. Versatile and open software for comparing large genomes. Genome Biol. 2004;5 2:R12. doi:10.1186/gb-2004-5-2-r12.
14. Walker BJ, Abeel T, Shea T, Priest M, Abouelliel A, Sakthikumar S, et al. Pilon: an integrated tool for comprehensive microbial variant detection and genome assembly improvement. Plos One. 2014;9 11:e112963. doi:10.1371/journal.pone.0112963.
15. Chang-Ming Bai, Lu-Sheng Xin, Umberto Rosani, Biao Wu, Qing-Chen Wang, Xiao-Ke Duan, et al. The pipeline of assembly and annotation of the *Scapharca broughtonii* genome. protocols.io. 2019; https://dx.doi.org/10.17504/protocols.io.z7zf9p6.
16. Li H, Handsaker B, Wysoker A, Fennell T, Ruan J, Homer N, et al. The Sequence Alignment/Map format and SAMtools. Bioinformatics. 2009;25 16:2078-9. doi:10.1093/bioinformatics/btp352.
17. Simao FA, Waterhouse RM, Ioannidis P, Kriventseva EV and Zdobnov EM. BUSCO: assessing genome assembly and annotation completeness with single-copy orthologs. Bioinformatics. 2015;31 19:3210-2. doi:10.1093/bioinformatics/btv351.
18. Li H. Aligning sequence reads, clone sequences and assembly contigs with BWA-MEM. arXiv. 2013;1303.3997.
19. Servant N, Varoquaux N, Lajoie BR, Viara E, Chen CJ, Vert JP, et al. HiC-Pro: an optimized and flexible pipeline for Hi-C data processing. Genome Biology. 2015;16 doi:ARTN 259
10.1186/s13059-015-0831-x.
20. Chang-Ming Bai, Lu-Sheng Xin, Umberto Rosani, Biao Wu, Qing-Chen Wang, Xiao-Ke Duan, et al. The pipeline of Hi-C assembly of the *Scapharca broughtonii* genome. protocols.io. 2019; https://dx.doi.org/10.17504/protocols.io.z8cf9sw.

21. Burton JN, Adey A, Patwardhan RP, Qiu R, Kitzman JO and Shendure J. Chromosome-scale scaffolding of de novo genome assemblies based on chromatin interactions. Nat Biotechnol. 2013;31 12:1119-25. doi:10.1038/nbt.2727.
22. Jay Ghurye, Mihai Pop, Sergey Koren, Derek Bickhart and Chin C-S. Scaffolding of long read assemblies using long range contact information. BMC Genomics. 2017;18:527.
23. Zhou L and Wang Z-C. Studies on karyotype analysis in the Scapharca broughtonii. Journal of Fisheries of China. 1997;21 4:455-7.
24. Xu Z and Wang H. LTR_FINDER: an efficient tool for the prediction of full-length LTR retrotransposons. Nucleic Acids Res. 2007;35 Web Server issue:W265-8. doi:10.1093/nar/gkm286.
25. Price AL, Jones NC and Pevzner PA. De novo identification of repeat families in large genomes. Bioinformatics. 2005;21 Suppl 1:i351-8. doi:10.1093/bioinformatics/bti1018.
26. Edgar RC and Myers EW. PILER: identification and classification of genomic repeats. Bioinformatics. 2005;21 Suppl 1:i152-8. doi:10.1093/bioinformatics/bti1003.
27. Wicker T, Sabot F, Hua-Van A, Bennetzen JL, Capy P, Chalhoub B, et al. A unified classification system for eukaryotic transposable elements. Nat Rev Genet. 2007;8 12:973-82. doi:10.1038/nrg2165.
28. Jurka J, Kapitonov VV, Pavlicek A, Klonowski P, Kohany O and Walichiewicz J. Repbase Update, a database of eukaryotic repetitive elements. Cytogenet Genome Res. 2005;110 1-4:462-7. doi:10.1159/000084979.
29. Tarailo-Graovac M and Chen N. Using RepeatMasker to identify repetitive elements in genomic sequences. Curr Protoc Bioinformatics. 2009;Chapter 4:Unit 4 10. doi:10.1002/0471250953.bi0410s25.
30. Burge C and Karlin S. Prediction of complete gene structures in human genomic DNA. J Mol Biol. 1997;268 1:78-94. doi:10.1006/jmbi.1997.0951.
31. Stanke M and Waack S. Gene prediction with a hidden Markov model and a new intron submodel. Bioinformatics. 2003;19 Suppl 2:ii215-25.
32. Majoros WH, Pertea M and Salzberg SL. TigrScan and GlimmerHMM: two open source ab initio eukaryotic gene-finders. Bioinformatics. 2004;20 16:2878-9. doi:10.1093/bioinformatics/bth315.
33. Blanco E, Parra G and Guigó R. Using geneid to identify genes. Current Protocols in Bioinformatics. 2007;18 1:4.3.1-4.3.28.
34. Korf I. Gene finding in novel genomes. BMC Bioinformatics. 2004;5:59. doi:10.1186/1471-2105-5-59.
35. Keilwagen J, Wenk M, Erickson JL, Schattat MH, Grau J and Hartung F. Using intron position conservation for homology-based gene prediction. Nucleic Acids Res. 2016;44 9:e89. doi:10.1093/nar/gkw092.
36. Bai CM, Rosani U, Xin LS, Li GY, Li C, Wang QC, et al. Dual transcriptomic analysis of Ostreid herpesvirus 1 infected *Scapharca broughtonii* with an emphasis on viral anti-apoptosis activities and host oxidative bursts. Fish Shellfish Immun. 2018;82:554-64. doi:10.1016/j.fsi.2018.08.054.
37. Campbell MA, Haas BJ, Hamilton JP, Mount SM and Buell CR. Comprehensive analysis of alternative splicing in rice and comparative analyses with Arabidopsis. BMC Genomics. 2006;7:327. doi:10.1186/1471-2164-7-327.
38. Pertea M, Kim D, Pertea GM, Leek JT and Salzberg SL. Transcript-level expression analysis of RNA-seq experiments with HISAT, StringTie and Ballgown. Nat Protoc. 2016;11 9:1650-67. doi:10.1038/nprot.2016.095.
39. Tang S, Lomsadze A and Borodovsky M. Identification of protein coding regions in RNA transcripts. Nucleic Acids Res. 2015;43 12:e78. doi:10.1093/nar/gkv227.
40. Haas BJ, Salzberg SL, Zhu W, Pertea M, Allen JE, Orvis J, et al. Automated eukaryotic gene structure annotation using EVidenceModeler and the Program to Assemble Spliced Alignments. Genome Biol. 2008;9 1:R7. doi:10.1186/gb-2008-9-1-r7.
41. Xiao J, Sekhwal MK, Li P, Ragupathy R, Cloutier S, Wang X, et al. Pseudogenes and Their Genome-Wide Prediction in Plants. International Journal of Molecular Sciences. 2016;17 12:1991.
42. Thibaud-Nissen F, Ouyang S and Buell CR. Identification and characterization of pseudogenes in the rice gene complement. BMC Genomics. 2009;10:317. doi:10.1186/1471-2164-10-317.
43. She R, Chu SC, Uyar B, Wang J, Wang K and Chen N. genBlastG: using BLAST searches to build homologous gene models. Bioinformatics. 2011;27 15:2141-3.
44. Birney E, Clamp M and Durbin R. GeneWise and Genomewise. Genome Res. 2004;14 5:988-95. doi:10.1101/gr.1865504.
45. Marchler-Bauer A, Lu S, Anderson JB, Chitsaz F, Derbyshire MK, DeWeese-Scott C, et al. CDD: a

Conserved Domain Database for the functional annotation of proteins. Nucleic Acids Res. 2011;39 Database issue:D225-9. doi:10.1093/nar/gkq1189.
46. Boeckmann B, Bairoch A, Apweiler R, Blatter MC, Estreicher A, Gasteiger E, et al. The SWISS-PROT protein knowledgebase and its supplement TrEMBL in 2003. Nucleic Acids Res. 2003;31 1:365-70.
47. Tatusov RL, Natale DA, Garkavtsev IV, Tatusova TA, Shankavaram UT, Rao BS, et al. The COG database: new developments in phylogenetic classification of proteins from complete genomes. Nucleic Acids Res. 2001;29 1:22-8.
48. Kanehisa M and Goto S. KEGG: kyoto encyclopedia of genes and genomes. Nucleic Acids Res. 2000;28 1:27-30.
49. Altschul SF, Gish W, Miller W, Myers EW and Lipman DJ. Basic local alignment search tool. J Mol Biol. 1990;215 3:403-10. doi:10.1016/S0022-2836(05)80360-2.
50. El-Gebali S, Mistry J, Bateman A, Eddy SR, Luciani A, Potter SC, et al. The Pfam protein families database in 2019. Nucleic Acids Res. 2018; doi:10.1093/nar/gky995.
51. Eddy SR, Mitchison G and Durbin R. Maximum discrimination hidden Markov models of sequence consensus. J Comput Biol. 1995;2 1:9-23. doi:10.1089/cmb.1995.2.9.
52. Dimmer EC, Huntley RP, Alam-Faruque Y, Sawford T, O'Donovan C, Martin MJ, et al. The UniProt-GO Annotation database in 2011. Nucleic Acids Res. 2012;40 Database issue:D565-70. doi:10.1093/nar/gkr1048.
53. Conesa A, Gotz S, Garcia-Gomez JM, Terol J, Talon M and Robles M. Blast2GO: a universal tool for annotation, visualization and analysis in functional genomics research. Bioinformatics. 2005;21 18:3674-6. doi:10.1093/bioinformatics/bti610.
54. Griffiths-Jones S, Moxon S, Marshall M, Khanna A, Eddy SR and Bateman A. Rfam: annotating non-coding RNAs in complete genomes. Nucleic Acids Res. 2005;33 Database issue:D121-4. doi:10.1093/nar/gki081.
55. Griffiths-Jones S, Grocock RJ, van Dongen S, Bateman A and Enright AJ. miRBase: microRNA sequences, targets and gene nomenclature. Nucleic Acids Res. 2006;34 Database issue:D140-4. doi:10.1093/nar/gkj112.
56. Nawrocki EP and Eddy SR. Infernal 1.1: 100-fold faster RNA homology searches. Bioinformatics. 2013;29 22:2933-5. doi:10.1093/bioinformatics/btt509.
57. Lowe TM and Eddy SR. tRNAscan-SE: a program for improved detection of transfer RNA genes in genomic sequence. Nucleic Acids Res. 1997;25 5:955-64.
58. Bai C; Xin L; Rosani U; Wu B; Wang Q; Duan X; Liu Z; Wang C (2019): Supporting data for "Chromosomal-level assembly of the blood clam, Scapharca (Anadara) broughtonii, using long sequence reads and Hi-C" GigaScience Database. http://dx.doi.org/10.5524/100607

Figure legends

Figure 1. Example of a *Scapharca* (*Anadara*) *broughtonii*, the blood clam.

Figure 2. Hi-C interaction heat map for *Scapharca* (*Anadara*) *broughtonii*.

Figure 3. Gene ontology (GO) annotation of the predicted genes.
The horizontal axis indicates classes of the second level GO annotation. The vertical axis indicates the number and percentage of genes in each class.

Figure 4. Eukaryotic Orthologous Groups (KOG) classification of the predicted genes.
Results are summarized in 24 function classes according to their functions. The horizontal axis represents each class, and the vertical axis represents the frequency of the classes.

Click here to access/download;Table;Table 1R.docx

Table 1. Summary of sequencing data generated for bloody clam genome assembly and annotation

Library type	Platform	Library size (bp)	Data size (Gb)	Application
Short reads	HiSeq X Ten	350	53.06	Genome survey, correction and evaluation
Long reads	PacBio SEQUEL	20,000	63.33	Genome assembly
	PacBio RS II	20,000	3.99	
	Nanopore Minion	20,000	8.47	
Hi-C	HiSeq X Ten	350	52.16	Chromosome construction

 Click here to access/download;Table;Table 2.docx

Table 2. Statics of the final genome assembly of *Scapharca* (*Anadara*) *broughtonii*

Types	Number	Length (bp)	N50 (bp)	N90 (bp)	Max (bp)	GC content	Gap (bp)
Scaffold	1026	884,566,040	44,995,656	25,444,477	55,667,740	33.70 %	65,100
Contig	1,667	884,500,940	1,797,717	305,905	7,852,409	33.70 %	0

 Click here to access/download;Table;Table 3r.docx

Table 3. Statics of gene annotation to different databases

Annotation database	Annotated number	Percentage (%)
GO_Annotation	5,766	23.98
KEGG_Annotation	9,174	38.15
KOG_Annotation	13,626	56.67
Pfam_Annotation	17,321	72.04
Swissprot_Annotation	12,866	53.51
TrEMBL_Annotation	21,887	91.03
nr_Annotation	21,897	91.07
nt_Annotation	12,786	53.18
All_Annotated	22,267	92.61

Figure 1 blood clam

Click here to access/download;Figure;Figure 1rcom.tif

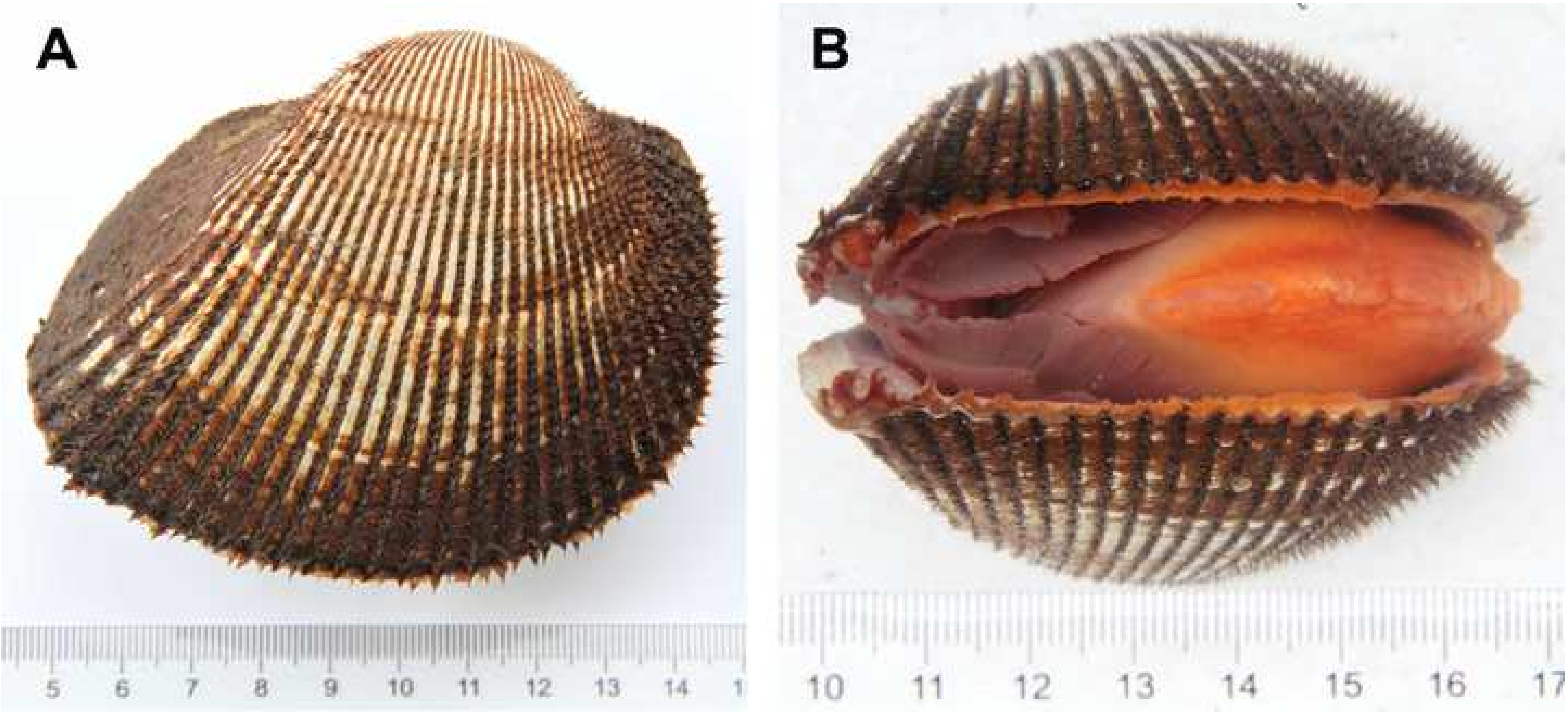

Figure 2

Click here to access/download;Figure;Figure1 Hi-C heatmap_for_final.jpeg

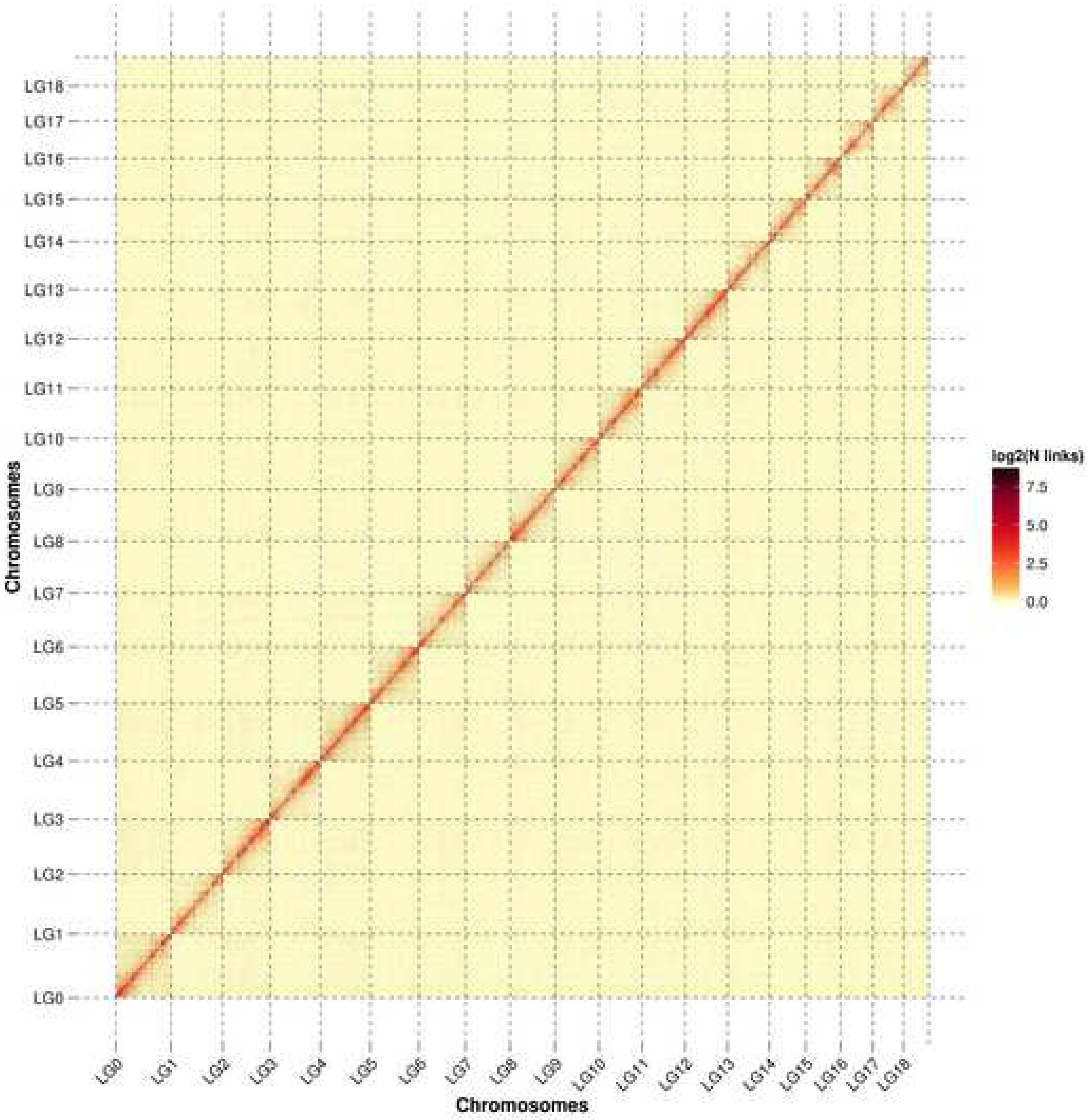

Figure 3

Click here to access/download;Figure;Figure2

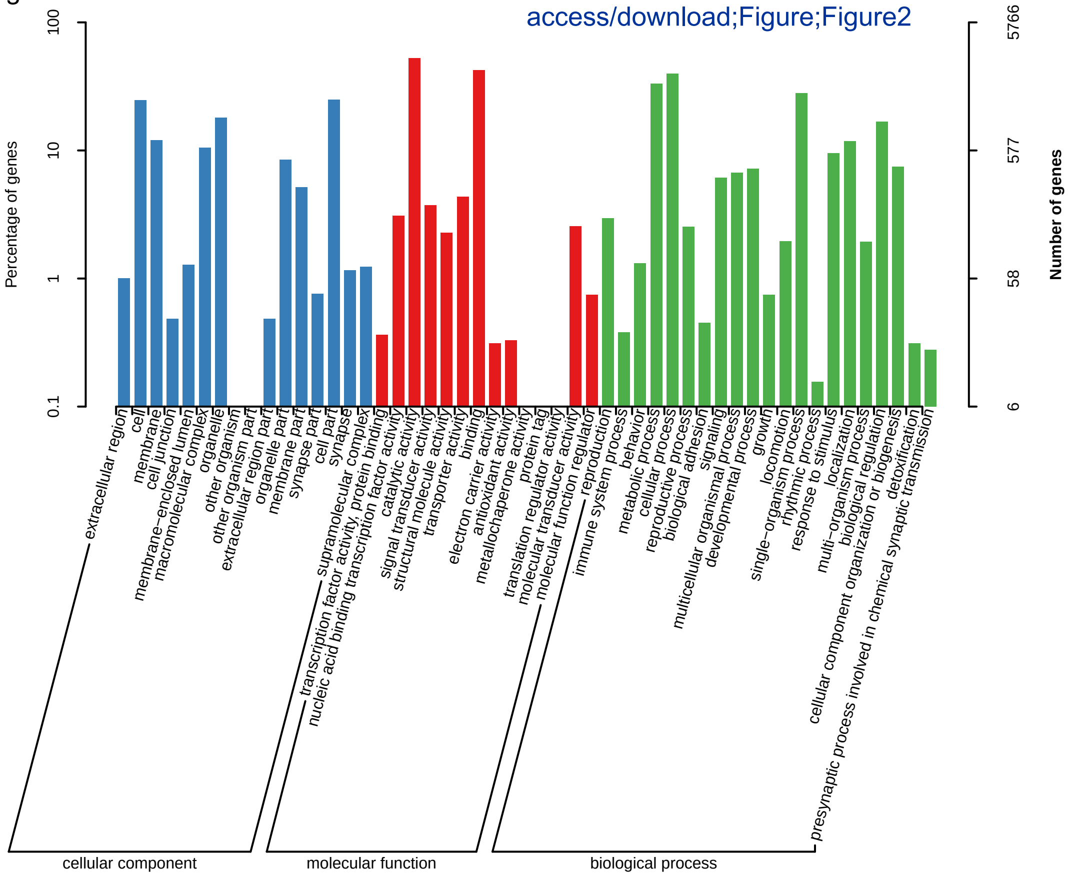

Figure 4

Click here to access/download;Figure;Figure3 EVM_GFF3.Kog.cluster.tif

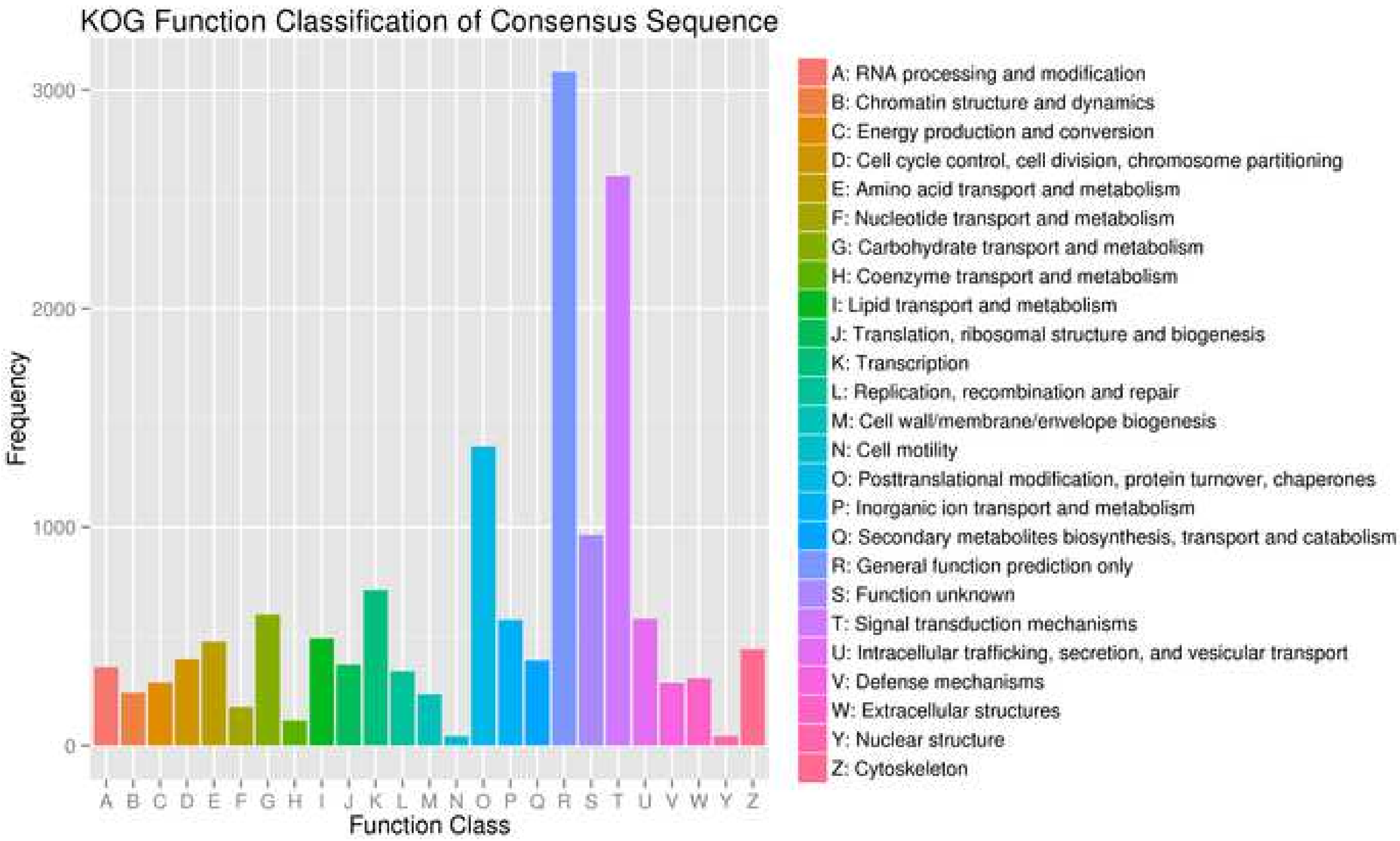

Supplementary Table S1

Click here to access/download
Supplementary Material
Supplementary Table1.xlsx

Click here to access/download
Supplementary Material
Supplementary material.docx

Supplementary Table S12

Click here to access/download
Supplementary Material
Supplementary Table12.xlsx

Personal Cover
Click here to access/download;Personal Cover;Cover letter-BCMr.docx

2019, Apr 21th

Dear GigaScience Editor,

We thank you and the two reviewers for the revision of our manuscript. We have read your requests/suggestions and those performed by the reviewers and each of these points have been revised carefully. We have added a figure (Figure 1) to show the shape and color of blood clam's shell and visceral mass. We have prepared a new supplementary table (the new Supplementary table 1) to present the key protocols, that were also uploaded to protocols.io as suggested by you. except that of Hi-C library preparation, We have provided more details of the key steps of Hi-C library construction at Lines 111-120. However, we do not include the very detailed Hi-C library protocol in Supplementary table 1, since it is a business secret of the BioMarker company, and they are still reluctant to provide us these parameters.

Here, we provide a point-by-point response to each reviewer's comments.

Sincerely,

Chong-Ming Wang

Yellow Sea Fisheries Research Institute (YSFRI)

E-mial: wangcm@ysfri.ac.cn

Reviewer #1

General comments:

Functional annotation was fairly extensive through the BLASTing of protein sequences to multiple databases. A statement should be added about the nr annotation as the nr database is not manually curated and is known to have errors that can be propogated. "Functional annotations that are found only in the nr database should not be used to annotate new genomes."

Following reviewer's comments, we have found and removed from the annotation table 41 genes annotated only in the Nr database. Now, we presented a final set of 22, 267 annotated genes.

The supplemental table with the blast annotations only contain the functional annotation without information about the blast score, length of alignment etc. It would be of great value to this data note if this information was added.

Following reviewer's comments, we have added blast score, length of alignment et al. for blast annotations to Nr and Nt databases. The detailed information regarding the functional annotations to

each database has been submitted to the GigaScience database (ftp://user95@parrot.genomics.cn) and it can be accessed by the reviewers using our credentials (user: user95 and password: WangCMClam). We prefer to not include all these details to the annotation supplemental table, since it will become difficult to read because of its large size.

Specific comments:

1) If available please state the number of places where Hi-C broke contigs in the assembly.

There are 343 broke points during the Hi-C scaffolding process, detailed information has been uploaded to GigaScience database (ftp://user95@parrot.genomics.cn). We have stated this point at Line 136 in the main text.

2) For all programs used please state the verson and all parameters required to replicate your analysis

We have provided the versions and parameters of all programs used in the manuscript at protocols.io and in the new Supplementary Table 1.

3) For all databases used (Kegg, nr KOG etc) please state the version or date of download used in annotation.

We have provided the version or date of download of all databases used in the manuscript at protocols.io and in the new Supplementary Table 1.

4) For the Blast analysis please specify if you used max-target-seq in your BLAST analysis and if you took the Best Blast Hit. How did you decide which Annotation to use?

Yes, we used the max-target-seq in our BLAST analysis with the parameter: -max_target_seqs 100 (we have specified this point at protocols.io and in the new Supplementary Table 1.). For the final annotation we have selected the annotation with the highest score

5) Please specify which Illumina reads were used during Pilon polishing.

The illumina reads for genome survey was used during Pilon polishing. We have stated this point at Line 98.

6) Would prefer that the authors include the blast result for each annotation provided in the supplemental table 11.

Please see the general comments.

Line 51: The word knew should be know. "Compared to oysters and scallops, we still know very little ..."

The section containing “knew” have been revised as a whole.

Reviewer #2

Major points:

The English of the manuscript is poor. In most places where there are issues, it is just awkward but in

some places the meaning is not clear.
We have invited a native speaker to kindly revise the language of the manuscript thoroughly. He fixed several language pitfalls and now we hope that the overall language quality is acceptable.
Was the DNA / material used all from the same individual?
We used haemocytes collected from several specimens for DNA extraction, to obtain enough DNA for the different libraries we constructed. We have specified this point in the main text at line 60.
How were the reads filtered (line 91)?
We used a custom perl script to filter the reads shorter than 500 bp. We have stated this point at Lines 93-94.
How many cycles of Pilon were used?
We used three cysles, we have stated this at lines 97-98.
What were the BUSCO results of the merged assembly before removal of redundancy with Numer? Were other tools such as Redundans explored for redundancy reduction?
We reduced the redundancy under the premise of keeping the integrity of the data. The evaluations of the different intermediate datasets were not included, while we displayed the best result. We did not use Redundans or other tools for redundancy reduction, since we were satisfied with the performance of Numer.
Methods used for Hi-C library preparation are inadequate.
We have provided more detailed information about the Hi-C library preparation at lines 111-120. We were not allowed to include some parameters because the protocol is a business secret of the BioMarker company, and they are reluctant to provide us these data.
The procedure described on lines 124-125 is not well explained. Why was this performed?
We have revised this section to provide more details and reasonability about Hi-C assembly at Lines 133-136.
Line 157: "the results of the three approaches" - unclear which three steps are referred to.
This refers to '*ab initio* prediction, homology-based prediction, and transcriptome-based prediction', We have revised this point at Line 168 to make it more clear.
Lines 160-161: The procedure to detect pseudogenes is not adequately described.
We have revise this section at Lines 171-177.
Availability of Data and Materials - what about the predicted transcripts and protein sequences?
These information has been uploaded to GigaScience database (ftp://user95@parrot.genomics.cn) and can be accessed by the reviewers using our credentials (see answer to Rew1), whereas they will immediately released after manuscript acceptance.

Minor points:
Line 38: To my knowledge, "ark shell" is a common name used for the entire family Arcidae, not just

this species.

We agree with you that "ark shell" is a common name used for the family Arcidae. The *Scapharca* (*Anadara*) *broughtonii* is always called ‘blood clam’ or ‘bloody clam’ in publications and Asia countries where the species is mainly distributed. So, we have revised this point indicating that ‘blood clam’ is a species of ‘ark shell’ (Lines 41-42).

Line 40: Correct "lived" to "lives"

We have replaced "lived" with "lives" at line 43.

Line 43: Correct "mollusk" to "molluscs"

We have replaced "mollusk" with "molluscs" at line 46.

Line 61: Correct "libraries" to "library"

We have replaced " libraries " with "library" at line 66.
