# Supplementary material for: Chromosomal-level assembly of the blood clam, Scapharca (Anadara) broughtonii, using long sequence reads and Hi-C
Source: Gigascience. 2019 Jul 9;8(7):giz067. doi: 10.1093/gigascience/giz067 (PMC6615981; doi:10.1093/gigascience/giz067)

**Supplementary material**

**For “Chromosomal-level assembly of the blood clam, *Scapharca* (*Anadara*) *broughtonii,* using long sequence reads and Hi-C”**

**Table S2 Summary of the Illumina sequencing reads used for genome survey, correction and evaluation.**

| Library | Data (Gb) | Depth (×) | Q20 (%) | Q30 (%) |
| --- | --- | --- | --- | --- |
| 350 bp | 53.06 | 61.49 | 97.32 | 94.08 |

**Table S3. Statics of the length distribution of Pacbio Subreads.**

| Length (bp) | Number | Total length (bp) | Average length (bp) |
| --- | --- | --- | --- |
| 500~2,000 | 548,413 | 669,509,974 | 1,220.81 |
| 2000~4,000 | 528,417 | 1,554,984,697 | 2,942.72 |
| 4000~6,000 | 425,303 | 2,115,625,339 | 4,974.40 |
| 6000~8,000 | 369,678 | 2,580,013,065 | 6,979.08 |
| 8000~10,000 | 336,146 | 3,021,399,974 | 8,988.36 |
| 10000~12,000 | 328,077 | 3,609,696,070 | 11,002.59 |
| 12000~14,000 | 322,843 | 4,194,052,108 | 12,991.00 |
| 14000~16,000 | 291,919 | 4,372,469,323 | 14,978.36 |
| 16000~18,000 | 249,029 | 4,225,849,636 | 16,969.31 |
| 18000~ | 1,361,272 | 40,916,556,273 | 30,057.59 |

**Table S4. Statics of the length distribution of Oxford Nanopore reads.**

| Length (bp) | Number | Total length (bp) | Average length (bp) |
| --- | --- | --- | --- |
| 500~2,000 | 42,158 | 51,692,569 | 1,226.16 |
| 2,000~4,000 | 47,156 | 139,891,753 | 2,966.57 |
| 4,000~6,000 | 40,069 | 199,248,307 | 4,972.63 |
| 6,000~8,000 | 35,519 | 248,082,723 | 6,984.51 |
| 8,000~10,000 | 34,657 | 312,347,543 | 9,012.54 |
| 10,000~12,000 | 41,146 | 454,116,798 | 11,036.72 |
| 12,000~14,000 | 45,796 | 595,707,844 | 13,007.86 |
| 14,000~16,000 | 44,764 | 670,935,480 | 14,988.28 |
| 16,000~18,000 | 39,728 | 674,349,441 | 16,974.16 |
| 18,000~ | 188,265 | 5,122,540,438 | 27,209.20 |

**Table S5. Statics of the initial genome assembly of *Scapharca* (*Anadara*) *broughtonii.***

| Contig number | Contig length (bp) | Contig N50 (bp) | Contig N90 (bp) | Contig max (bp) | GC content | Gap total length (bp) |
| --- | --- | --- | --- | --- | --- | --- |
| 1,334 | 884,500,940 | 2,388,811 | 581,086 | 19,384,884 | 33.70 % | 0 |

**Table S6. Summary of BUSCO analysis results.**

| Dataset | Complete BUSCOs | Complete and single-copy BUSCOs | Complete and duplicated BUSCOs | Fragmented BUSCOs | Missing BUSCOs |
| --- | --- | --- | --- | --- | --- |
| Eukaryote | 273 | 257 | 16 | 5 | 25 |
| Metazoan | 897 | 857 | 40 | 11 | 70 |

**Table S7. Statistics of the mapping results of Hi-C reads.**

| Mapping Type | Number | Ratio (%) |
| --- | --- | --- |
| Total Read Pairs | 174,148,156 | 100 |
| Mapped Reads | 206,299,359 | 59.23 |
| Unique Mapped Read Pairs | 51,075,780 | 29.33 |

**Table S8. Statistics of different types of the Hi-C reads.**

| Type | Number | Ratio (%) |
| --- | --- | --- |
| Unique Paired Alignments | 51,075,780 | 100 |
| Valid Interaction Pairs | 17,191,914 | 33.66 |
| Dangling End Pairs | 25,524,027 | 49.97 |
| Re-ligation Pairs | 2,258,899 | 4.42 |
| Self-cycle Pairs | 123,262 | 0.24 |
| Dumped Pairs | 5,977,678 | 11.7 |

**Table S9. Summary of the Hi-C assembly.**

| Group | Sequence Number | Sequence Length (bp) |
| --- | --- | --- |
| Lachesis Group0 | 88 | 60,120,809 |
| Lachesis Group1 | 107 | 57,716,977 |
| Lachesis Group2 | 83 | 54,700,034 |
| Lachesis Group3 | 54 | 52,314,689 |
| Lachesis Group4 | 43 | 51,429,223 |
| Lachesis Group5 | 53 | 50,608,661 |
| Lachesis Group6 | 78 | 50,388,922 |
| Lachesis Group7 | 103 | 49,063,309 |
| Lachesis Group8 | 74 | 48,667,129 |
| Lachesis Group9 | 65 | 46,858,284 |
| Lachesis Group10 | 78 | 46,427,082 |
| Lachesis Group11 | 63 | 45,526,693 |
| Lachesis Group12 | 67 | 45,029,501 |
| Lachesis Group13 | 78 | 44,995,855 |
| Lachesis Group14 | 76 | 39,900,971 |
| Lachesis Group15 | 69 | 37,995,990 |
| Lachesis Group16 | 75 | 34,958,828 |
| Lachesis Group17 | 60 | 33,302,253 |
| Lachesis Group18 | 70 | 28,784,282 |
| Total Sequences  Clustered (Ratio %) | 1,384(82.53) | 878,789,492(99.35) |
| Total Sequences Ordered and  Oriented (Ratio %) | 670(48.41) | 819,165,139(93.22) |

**Table S10. Statistics of the repeated sequences.**

| Type | Number | Length（bp） | Percentage (%) |
| --- | --- | --- | --- |
| ClassI/DIRS | 1,343 | 2,044,912 | 0.23 |
| ClassI/LINE | 7,654 | 14,606,373 | 1.65 |
| ClassI/LTR | 299 | 560,299 | 0.06 |
| ClassI/LTR/Copia | 148 | 196,974 | 0.02 |
| ClassI/LTR/Gypsy | 4,649 | 8,298,938 | 0.94 |
| ClassI/PLE\|LARD | 449,730 | 100,185,783 | 11.33 |
| ClassI/SINE | 5,815 | 925,217 | 0.1 |
| ClassI/SINE\|TRIM | 3 | 5,048 | 0 |
| ClassI/TRIM | 103,314 | 35,450,675 | 4.01 |
| ClassI/Unknown | 746 | 77,849 | 0.01 |
| ClassII/Crypton | 10,563 | 2,385,627 | 0.27 |
| ClassII/Helitron | 85,556 | 19,790,836 | 2.24 |
| ClassII/MITE | 35,971 | 9,235,728 | 1.04 |
| ClassII/Maverick | 5,163 | 993,226 | 0.11 |
| ClassII/TIR | 86,638 | 33,366,573 | 3.77 |
| ClassII/Unknown | 63,717 | 12,658,075 | 1.43 |
| PotentialHostGene | 120,712 | 21,046,647 | 2.38 |
| SSR | 8,413 | 2,523,616 | 0.29 |
| Unknown | 1,232,408 | 240,659,216 | 27.21 |
| Total | 2,222,842 | 407,797,738 | 46.1 |

**Table S11. Summary of the gene prediction results.**

| Method | Software | Species | Gene number |
| --- | --- | --- | --- |
| *Ab initio* | Genscan | / | 23,354 |
|  | Augustus | / | 34,510 |
|  | GlimmerHMM | / | 23,946 |
|  | GeneID | / | 24,893 |
|  | SNAP | / | 37,567 |
| Homology-based | GeMoMa | *Danio rerio* | 11,570 |
|  |  | *Crassostrea gigas* | 21,507 |
|  |  | *Mizuhopecten yessoensis* | 22,454 |
|  |  | *Mytilus galloprovincialis* | 4,393 |
| RNA-seq | PASA | / | 3,701 |
|  | GeneMarkS-T | / | 15,940 |
|  | TransDecoder | / | 21,782 |
| Integration | EVM | / | 24,045 |

**Figure S1. Species distribution of BLAST hits of the predicted genes in the NR database.**


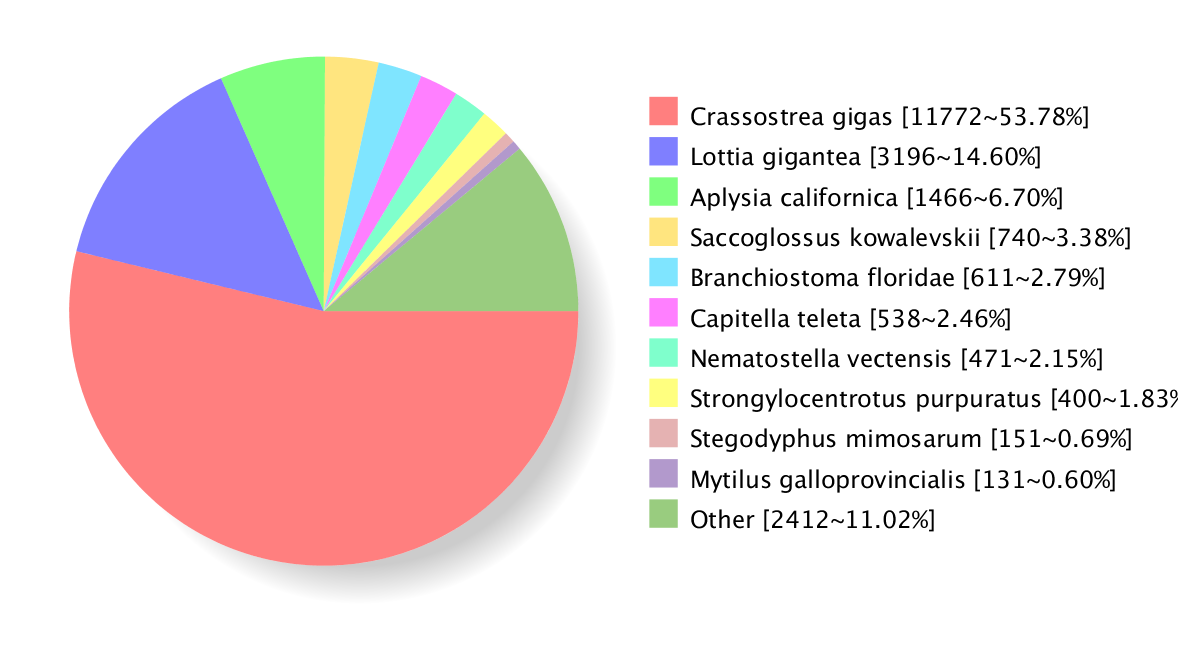

Supplement: giz067_Supplemental_Files [file giz067_supplemental_files.zip › Supplementary material.docx]
