# Supplementary material for: Chromosomal-level assembly of the blood clam, Scapharca (Anadara) broughtonii, using long sequence reads and Hi-C
Source: Gigascience. 2019 Jul 9;8(7):giz067. doi: 10.1093/gigascience/giz067 (PMC6615981; doi:10.1093/gigascience/giz067)
Supplement: giz067_Response_to_Reviewer_Comments_Original_Submission [file giz067_response_to_reviewer_comments_original_submission.pdf]

Author's Response To Reviewer Comments

Close

Dear GigaScience Editor,
We thank you and the two reviewers for the revision of our manuscript. We have read your requests/suggestions and those performed by the reviewers and each of these points have been revised carefully. We have added a figure (Figure 1) to show the shape and color of blood clam's shell and visceral mass. We have prepared a new supplementary table (the new Supplementary table 1) to present the key protocols, that were also uploaded to protocols.io as suggested by you. except that of Hi-C library preparation, We have provided more details of the key steps of Hi-C library construction at Lines 111-120. However, we do not include the very detailed Hi-C library protocol in Supplementary table 1, since it is a business secret of the BioMarker company, and they are still reluctant to provide us these parameters.
Here, we provide a point-by-point response to each reviewer's comments.

Sincerely,
Chong-Ming Wang
Yellow Sea Fisheries Research Institute (YSFRI)
E-mial: wangcm@ysfri.ac.cn

Reviewer #1
General comments:
Functional annotation was fairly extensive through the BLASTing of protein sequences to multiple databases. A statement should be added about the nr annotation as the nr database is not manually curated and is known to have errors that can be propogated. "Functional annotations that are found only in the nr database should not be used to annotate new genomes."
Following reviewer's comments, we have found and removed from the annotation table 41 genes annotated only in the Nr database. Now, we presented a final set of 22, 267 annotated genes.
The supplemental table with the blast annotations only contain the functional annotation without information about the blast score, length of alignment etc. It would be of great value to this data note if this information was added.
Following reviewer's comments, we have added blast score, length of alignment et al. for blast annotations to Nr and Nt databases. The detailed information regarding the functional annotations to each database has been submitted to the GigaScience database (ftp://user95@parrot.genomics.cn) and it can be accessed by the reviewers using our credentials (user: user95 and password: WangCMClam). We prefer to not include all these details to the annotation supplemental table, since it will become difficult to read because of its large size.
Specific comments:
1) If available please state the number of places where Hi-C broke contigs in the assembly.
There are 343 broke points during the Hi-C scaffolding process, detailed information has been uploaded to GigaScience database (ftp://user95@parrot.genomics.cn). We have stated this point at Line 136 in the main text.
2) For all programs used please state the verson and all parameters required to replicate your analysis
We have provided the versions and parameters of all programs used in the manuscript at protocols.io and in the new Supplementary Table 1.
3) For all databases used (Kegg, nr KOG etc) please state the version or date of download used in annotation.
We have provided the version or date of download of all databases used in the manuscript at protocols.io and in the new Supplementary Table 1.
4) For the Blast analysis please specify if you used max-target-seq in your BLAST analysis and if you took the Best Blast Hit. How did you decide which Annotation to use?
Yes, we used the max-target-seq in our BLAST analysis with the parameter: -max_target_seqs 100 (we have specified this point at protocols.io and in the new Supplementary Table 1.). For the final annotation we have selected the annotation with the highest score
5) Please specify which Illumina reads were used during Pilon polishing.
The illumina reads for genome survey was used during Pilon polishing. We have stated this point at Line

98.
6) Would prefer that the authors include the blast result for each annotation provided in the supplemental table 11.
Please see the general comments.
Line 51: The word knew should be know. "Compared to oysters and scallops, we still know very little ..."
The section containing "knew" have been revised as a whole.

Reviewer #2
Major points:
The English of the manuscript is poor. In most places where there are issues, it is just awkward but in some places the meaning is not clear.
We have invited a native speaker to kindly revise the language of the manuscript thoroughly. He fixed several language pitfalls and now we hope that the overall language quality is acceptable.
Was the DNA / material used all from the same individual?
We used haemocytes collected from several specimens for DNA extraction, to obtain enough DNA for the different libraries we constructed. We have specified this point in the main text at line 60.
How were the reads filtered (line 91)?
We used a custom perl script to filter the reads shorter than 500 bp. We have stated this point at Lines 93-94.
How many cycles of Pilon were used?
We used three cysles, we have stated this at lines 97-98.
What were the BUSCO results of the merged assembly before removal of redundancy with Numer? Were other tools such as Redundans explored for redundancy reduction?
We reduced the redundancy under the premise of keeping the integrity of the data. The evaluations of the different intermediate datasets were not included, while we displayed the best result. We did not use Redundans or other tools for redundancy reduction, since we were satisfied with the performance of Numer.
Methods used for Hi-C library preparation are inadequate.
We have provided more detailed information about the Hi-C library preparation at lines 111-120. We were not allowed to include some parameters because the protocol is a business secret of the BioMarker company, and they are reluctant to provide us these data.
The procedure described on lines 124-125 is not well explained. Why was this performed?
We have revised this section to provide more details and reasonability about Hi-C assembly at Lines 133-136.
Line 157: "the results of the three approaches" - unclear which three steps are referred to.
This refers to 'ab initio prediction, homology-based prediction, and transcriptome-based prediction', We have revised this point at Line 168 to make it more clear.
Lines 160-161: The procedure to detect pseudogenes is not adequately described.
We have revise this section at Lines 171-177.
Availability of Data and Materials - what about the predicted transcripts and protein sequences?
These information has been uploaded to GigaScience database (ftp://user95@parrot.genomics.cn) and can be accessed by the reviewers using our credentials (see answer to Rew1), whereas they will immediately released after manuscript acceptance.

Minor points:
Line 38: To my knowledge, "ark shell" is a common name used for the entire family Arcidae, not just this species.
We agree with you that "ark shell" is a common name used for the family Arcidae. The Scapharca (Anadara) broughtonii is always called 'blood clam' or 'bloody clam' in publications and Asia countries where the species is mainly distributed. So, we have revised this point indicating that 'blood clam' is a species of 'ark shell' (Lines 41-42).
Line 40: Correct "lived" to "lives"
We have replaced "lived" with "lives" at line 43.
Line 43: Correct "mollusk" to "molluscs"
We have replaced "mollusk" with "molluscs" at line 46.
Line 61: Correct "libraries" to "library"
We have replaced " libraries " with "library" at line 66.

Close
