# Supplementary material for: Chromosomal-level assembly of the blood clam, Scapharca (Anadara) broughtonii, using long sequence reads and Hi-C
Source: Gigascience. 2019 Jul 9;8(7):giz067. doi: 10.1093/gigascience/giz067 (PMC6615981; doi:10.1093/gigascience/giz067)
Supplement: giz067_Reviewer_1_Report_Original_Submission — Andrew Severin -- 3/15/2019 Reviewed [file giz067_reviewer_1_report_original_submission.pdf]

Reviewer Report

Title: Chromosomal-level assembly of the blood clam, Scapharca (Anadara) broughtonii, using long sequence reads and Hi-C

Version: Original Submission **Date:** 3/15/2019

Reviewer name: Andrew Severin

Reviewer Comments to Author:

This data note describes the genome assembly and annotation of the bloody clam Scapharca (Anadara) broughtonii. Bloody clam or ark shell is an important aquaculture species and is known for its red color in their visceral mass due to hemoglobin which is not typical of Mollusks.
Data from PacBio and Nanopore sequencing was performed to an approximate level of 86x coverage for the genome. Assembly was performed using Canu (v1.5) and WTDBG, Quickmerge to merge the asemblies and then Numer to remove redundancy. Pilon was used to polish the assembly with Illumina reads.
Hi-C data was also generated and used to scaffold the contigs into 19 chromosomes. Scaffolding was performed using Lachesis which is the precursor to Juicer. Based on the plots, the final assembly looks good. It would be nice if the authors stated how many times the contigs in their initial assembly was broken during the Hi-C scaffolding process due to missassemblies produced from the canu, wtdbg and quickmerge process.
Gene model annotation was perfromed using ab initio (Augustus, GlimmerHMM, GeneID, SNAP) and homology based prediction using (Crassostrea gigas, Mizuhopecten yessoensis and Mytilus galloprovincincialis and Danio Rerio). All gene model information was integerated using EVM and polished with PASA. Note to Authors that the Mytilus galloprovincincialis is a really bad assembly and may not have been the best choice for using gene models. However, given the wealth of other transcriptomic data used, I don't anticipate this had a significant impact on the quality of the gene models predicted.
Functional annotation was fairly extensive through the BLASTing of protein sequences to multiple databases. A statement should be added about the nr annotation as the nr database is not manually curated and is known to have errors that can be propogated. "Functional annotations that are found only in the nr database should not be used to annotate new genomes."
The supplamental table with the blast annotations only contain the functional annotation without information about the blast score, length of alignment etc. It would be of great value to this data note if this information was added.
Overall, a nice data note with a good looking genome assembly. Glad to see another mollusk genome assembly.

1) If available please state the number of places where Hi-C broke contigs in the assembly.
2) For all programs used please state the verson and all parameters required to replicate your analysis
3) For all databases used (Kegg, nr KOG etc) please state the version or date of download used in

annotation.
4) For the Blast analysis please specify if you used max-target-seq in your BLAST analysis and if you took the Best Blast Hit. How did you decide which Annotation to use?
5) Please specify which Illumina reads were used during Pilon polishing.
6) Would prefer that the authors include the blast result for each annotation provided in the supplemental table 11.
Line 51: The word knew should be know. "Compared to oysters and scallops, we still know very little ..."

Level of Interest

Please indicate how interesting you found the manuscript: Choose an item.

Quality of Written English

Please indicate the quality of language in the manuscript: Choose an item.

Declaration of Competing Interests

Please complete a declaration of competing interests, considering the following questions:

- Have you in the past five years received reimbursements, fees, funding, or salary from an organisation that may in any way gain or lose financially from the publication of this manuscript, either now or in the future?
- Do you hold any stocks or shares in an organisation that may in any way gain or lose financially from the publication of this manuscript, either now or in the future?
- Do you hold or are you currently applying for any patents relating to the content of the manuscript?
- Have you received reimbursements, fees, funding, or salary from an organization that holds or has applied for patents relating to the content of the manuscript?
- Do you have any other financial competing interests?
- Do you have any non-financial competing interests in relation to this paper?

If you can answer no to all of the above, write 'I declare that I have no competing interests' below. If your reply is yes to any, please give details below.

none

I agree to the open peer review policy of the journal. I understand that my name will be included on my report to the authors and, if the manuscript is accepted for publication, my named report including any attachments I upload will be posted on the website along with the authors' responses. I agree for my report to be made available under an Open Access Creative Commons CC-BY license (http://creativecommons.org/licenses/by/4.0/). I understand that any comments which I do not wish to

be included in my named report can be included as confidential comments to the editors, which will not be published.

I agree to the open peer review policy of the journal

To further support our reviewers, we have joined with Publons, where you can gain additional credit to further highlight your hard work (see: https://publons.com/journal/530/gigascience). On publication of this paper, your review will be automatically added to Publons, you can then choose whether or not to claim your Publons credit. I understand this statement.

Yes Choose an item.
