# Supplementary material for: Chromosomal-level assembly of the blood clam, Scapharca (Anadara) broughtonii, using long sequence reads and Hi-C
Source: Gigascience. 2019 Jul 9;8(7):giz067. doi: 10.1093/gigascience/giz067 (PMC6615981; doi:10.1093/gigascience/giz067)
Supplement: giz067_Reviewer_2_Report_Original_Submission — Kevin Kocot -- 3/16/2019 Reviewed [file giz067_reviewer_2_report_original_submission.pdf]

Reviewer Report

Title: Chromosomal-level assembly of the blood clam, Scapharca (Anadara) broughtonii, using long sequence reads and Hi-C

Version: Original Submission **Date:** 3/16/2019

Reviewer name: Kevin Kocot

Reviewer Comments to Author:

This data note describes the chromosome-level assembly of the bivalve mollusc Scapharca broughtonii. This is an economically important species and this genome will represent the first from the clade Arcidae. The genome assembly appears to be very high quality and this should be useful resource. Howeer, there are a number of revisions that I feel need to be made before this manuscript is ready for publication.
The English of the manuscript is poor. In most places where there are issues, it is just awkward but in some places the meaning is not clear.
Major points:
Was the DNA / material used all from the same individual?
How were the reads filtered (line 91)?
How many cycles of Pilon were used?
What were the BUSCO results of the merged assembly before removal of redundancy with Numer?
Were other tools such as Redundans explored for redundancy reduction?
Methods used for Hi-C library preparation are inadequate.
The procedure described on lines 124-125 is not well explained. Why was this performed?
Line 157: "the results of the three approaches" - unclear which three steps are referred to.
Lines 160-161: The procedure to detect pseudogenes is not adequately described.
Availability of Data and Materials - what about the predicted transcripts and protein sequences?
Minor points:
Line 38: To my knowledge, "ark shell" is a common name used for the entire family Arcidae, not just this species.
Line 40: Correct "lived" to "lives"
Line 43: Correct "mollusk" to "molluscs"
Line 61: Correct "libraries" to "library"

Level of Interest

Please indicate how interesting you found the manuscript: Choose an item.

Quality of Written English

Please indicate the quality of language in the manuscript: Choose an item.

Declaration of Competing Interests

Please complete a declaration of competing interests, considering the following questions:

- Have you in the past five years received reimbursements, fees, funding, or salary from an organisation that may in any way gain or lose financially from the publication of this manuscript, either now or in the future?
- Do you hold any stocks or shares in an organisation that may in any way gain or lose financially from the publication of this manuscript, either now or in the future?
- Do you hold or are you currently applying for any patents relating to the content of the manuscript?
- Have you received reimbursements, fees, funding, or salary from an organization that holds or has applied for patents relating to the content of the manuscript?
- Do you have any other financial competing interests?
- Do you have any non-financial competing interests in relation to this paper?

If you can answer no to all of the above, write 'I declare that I have no competing interests' below. If your reply is yes to any, please give details below.

I declare that I have no competing interests.

I agree to the open peer review policy of the journal. I understand that my name will be included on my report to the authors and, if the manuscript is accepted for publication, my named report including any attachments I upload will be posted on the website along with the authors' responses. I agree for my report to be made available under an Open Access Creative Commons CC-BY license (http://creativecommons.org/licenses/by/4.0/). I understand that any comments which I do not wish to be included in my named report can be included as confidential comments to the editors, which will not be published.

I agree to the open peer review policy of the journal

To further support our reviewers, we have joined with Publons, where you can gain additional credit to further highlight your hard work (see: https://publons.com/journal/530/gigascience). On publication of this paper, your review will be automatically added to Publons, you can then choose whether or not to claim your Publons credit. I understand this statement.

Yes Choose an item.
